# Species delimitation in the *Populus laurifolia* complex (Salicaceae) based on phylogenetic and morphometric evidence

**DOI:** 10.3389/fpls.2025.1518122

**Published:** 2025-02-06

**Authors:** Xueya Wei, Xingyong Cui, Fulin Yuan, Kerou Zhou, Liwei Zhou, Changli Zhao, Shaoyu Guo, Ce Shang, Zhixiang Zhang

**Affiliations:** Laboratory of Systematic Evolution and Biogeography of Woody Plants, School of Ecology and Nature Conservation, Beijing Forestry University, Beijing, China

**Keywords:** *Populus laurifolia*, species complex, phylogenomics, species delimitation, integrative taxonomy

## Abstract

Due to significant morphological differences and extensive interspecific hybridization, there are numerous species complexes with taxonomic challenges in the genus *Populus*. Integrative taxonomy, which combines evidence of morphology, molecular phylogeny, niche differentiation, and reproductive isolation, provides the most effective approaches for species delimitation. The *Populus laurifolia* complex, which belongs to *Populus* subg. *Tacamahaca* (Salicaceae), is distributed in the Altai Mountains and Tianshan Mountains. This complex exhibits morphological variability, making species delimitation challenging. Due to limited sampling and systematic studies, its taxonomy has remained unresolved. In this study, 337 specimens, along with online digital samples representing nearly all wild populations, were collected. Morphological analyses were performed to evaluate key traits and clarify species boundaries. Phylogenetic relationships were reconstructed using concatenation and coalescent methods based on 566,375 nuclear single-nucleotide polymorphisms (SNPs). Ecological niche differentiation was assessed, and ABBA–BABA analysis was used to examine interspecific hybridization. The results revealed that this complex, based on a series of significant character states, could be morphologically distinguished into three species—*P. laurifolia* (*Populus pilosa* considered a synonym of *P. laurifolia*), *Populus talassica*, and *Populus pamirica*—which also correspond to three well-supported clades in the phylogenetic trees. *P. pamirica* exhibits some degree of ecological niche differentiation from *P. talassica* and *P. laurifolia*, whereas the latter two show minimal differentiation. Gene flow within the complex remains limited. This research underscores the importance of integrating multiple lines of evidence in the classification of *Populus*, providing a framework for future taxonomic studies.

## 1 Introduction

Accurate delimitation of species is essential first of all for its correct taxonomic identification and then also to evaluate biodiversity, even for species within the genus *Populus* L. (Sites and Crandall, 1997; Yang and Rannala, 2010). Relying solely on a single method for species classification, such as morphology, may introduce bias and lead to the failure to recognize cryptic species or result in overclassification (Liu, 2016; Wan et al., 2023). The genus *Populus* (Salicaceae), commonly known as poplars, is widely distributed in the northern hemisphere throughout the subtropical to boreal forests and is well-known for its economic, ecological, and evolutionary importance as pioneer species (Braatne et al., 1992). However, classifying *Populus* is particularly challenging due to extensive hybridization and genetic introgression throughout evolution and speciation, which can obscure interspecific boundaries (Harland, 1950; Jiang et al., 2016; Wang et al., 2020; Du et al., 2024). Traditionally, the classification of *Populus* relies on morphological characteristics. However, this approach often struggles to account for the complex hybridization patterns within the genus. For instance, the *Flora of China* (Fang et al., 1999) recognized 71 species, with some considered varieties or hybrids of more diagnostic species, while Eckenwalder’s (1996) system identified only 29 species all over the world, highlighting the discrepancies in species delimitation based on morphology alone.

To clarify ambiguous species boundaries and reduce overclassification, the integrative species concept (Liu, 2016) employed multiple criteria for species classification. This approach incorporates various lines of evidence, including phenotypic distinction, phylogenetic monophyly (Donoghue, 1985; Minh et al., 2013), niche differentiation, and reproductive isolation (Coyne et al., 1988). Geographic isolation can be considered alongside niche differentiation as complementary evidence for species delimitation (Maya-Lastra et al., 2024). The use of multiple lines of evidence enhanced the objectivity and accuracy of species delimitation and taxonomic revision, particularly in identifying cryptic species and defining composite species (Feng et al., 2016; Liu, 2016; Zhang et al., 2018b; Lu et al., 2021; Perrino et al., 2022). Morphological data served as the foundation of taxonomic studies, while genetic evidence increasingly uncovered diversity not detected through morphology alone (Hu et al., 2018; Li et al., 2019; Sassone et al., 2021). Early molecular markers often lacked sufficient informative loci, limiting their ability to accurately reflect interspecies relationships (Castiglione et al., 1993; Bradshaw et al., 1994; Cervera et al., 2005; Feng et al., 2013; Wan et al., 2023). Chloroplast genomes, which reflect only maternal inheritance, also failed to differentiate species, particularly in taxa that exhibit extensive hybridization and chloroplast capture (Han et al., 2017; Zhang et al., 2018a; Zhou et al., 2021; Wang et al., 2022). Whole-genome resequencing offers a comprehensive view of the genomic landscape, overcoming the limitations of earlier methods and providing a more accurate assessment of phylogenetic relationships (Wang et al., 2022; Yang et al., 2022). Therefore, applying whole-genome resequencing is crucial in accurately deciphering the phylogenetic relationships of the *Populus* species and resolving taxonomic uncertainties. The *Populus laurifolia* Ledeb. complex is primarily distributed across northern and central Asia and has been established as monophyletic (Wang et al., 2022). This complex encompasses one to four recognized species. The *Flora of the U.S.S.R.* (Komarov, 1936) and *Flora of China* (Fang et al., 1999) reported four species: *P. laurifolia*, *Populus pilosa* Rehd., *Populus pamirica* Kom., and *Populus talassica* Kom. Additionally, *Flora of Xinjiangensis* (Yang, 1993) includes three varieties under these species: *P. talassica* var. *cordata* C.Y. Yang, *P. talassica* var. *tomortensis* C.Y. Yang, and *P. pamirica* var. *akqiensis* C.Y. Yang. *Flora Reipublicae Popularis Sinicae* (*FRPS*; Wang and Fang, 1984) included another variant, *P. pilosa* var. *leiocarpa* C. Wang & S. L. Tung. In contrast, the North American system reported only *P. laurifolia* (Eckenwalder, 1977, 1996). In this study, we included all aforementioned species within the *P. laurifolia* complex.

*Populus laurifolia* is widely recognized as a separate species based on various morphological and phylogenetic studies (Skvortsov, 2009, 2010; Wang et al., 2020; Wang et al., 2022). Specifically, *P. pilosa* shares several characteristics with *P. laurifolia*, including similar growth habits, habitat preference (riverbanks and wetlands), ovate to elliptic leaves with a hairy texture, and hairy fruits. These similarities often lead to the misidentification of *P. pilosa* as *P. laurifolia*. The primary distinction lies in the leaf base of *P. pilosa*, which is cordate or slightly cordate. *Populus pilosa* has recently been treated as a synonym or an infraspecific variant of *Populus laurifolia* (Skvortsov, 2010). As stated in the original literature (Wang and Tung, 1982), *P. pilosa* var. *leiocarpa* is distinguished solely by its glabrous fruits and was described as a variety of *P. pilosa*. Since its initial description, no comprehensive phylogenetic studies have been conducted on *P. pilosa* and its variety, leaving their taxonomic positions unresolved. The absence of phylogenetic data raises doubts about their relationships with *P. laurifolia*, underscoring the necessity for further molecular analyses. Morphological similarities also exist among *P. laurifolia*, *P. talassica*, and *P. pamirica*. *P. talassica* is primarily distinguished from *P. laurifolia* by its almost glabrous petioles and fruits, as well as its nearly non-angular sprouts. In contrast, *P. pamirica* can be distinguished from *P. laurifolia* by the aspect ratios of its leaf blades, which are nearly equal. *Flora of Kazakhstan* (Baitenov, 2001) considered *P. talassica* synonymous with *Populus cathayana* Rehd. due to characteristics such as short branches and petioles that are initially hairy but become glabrous upon maturation. This viewpoint has been widely rejected by many scholars (Yang, 1993). Recent studies also revealed that *P. talassica* is more closely related to *P. pamirica*, forming a sister group (Wang et al., 2020; Wang et al., 2022). Komarov (1936) initially described these two species with *Populus densa* Kom. for Central Asia, also referenced in the *Flora of the U.S.S.R.* Skvortsov (1972) suggested that *P. talassica* and *P. pamirica* refer to the same species, subsequently naming *P. talassica*. In contrast, *Flora of Xinjiangensis* (Yang, 1993) rejected the notion that *P. pamirica* is a synonym of *P. talassica*. In 2011, Skvortsov merged *P. pamirica*, *P. talassica*, *P. densa*, *Populus suaveolens* var. *macrocarpa* Schrenk, and *P. cathayana* into *Populus macrocarpa* (Schrenk) Pavlov & Lipsch (Skvortsov, 2011). Recent molecular phylogenetic studies suggested otherwise, indicating that *P. cathayana*, *Populus shanxiensis* C. Wang & S. L. Tung, and *Populus intramongolica* T. Y. Sun & E. W. Ma formed a distinct monophyletic group, while *P. suaveolens* Fisch. clustered with *Populus maximowiczii* A. Henry, *Populus koreana* Rehder, and *Populus ussuriensis* Kom (Wang et al., 2014, 2020, 2022). These findings raised doubts about Skvortsov’s classification, indicating that it lacked phylogenetic support.

The morphological similarity among taxa within the *P. laurifolia* complex has created significant ambiguity regarding interspecific boundaries, leading to a wide range of opinions on species delimitation. These assumptions have been primarily based on morphological evidence, without robust phylogenetic studies to elucidate the species boundary of the *P. laurifolia* complex. Due to early molecular techniques and incomplete sampling, previous phylogenetic studies have left the interspecific relationships within the *P. laurifolia* complex unresolved (Feng et al., 2013; Wang et al., 2020; Wang et al., 2022). Aiming to clarify the species delimitation of the *P. laurifolia* complex, this study relies on comprehensive analyses of morphological traits, niche differentiation, and phylogenomic analyses using whole-genome resequencing and seeks to provide a clearer understanding of the interspecific relationships within this complex.

## 2 Materials and methods

### 2.1 Sample collection

**Figure 1:**
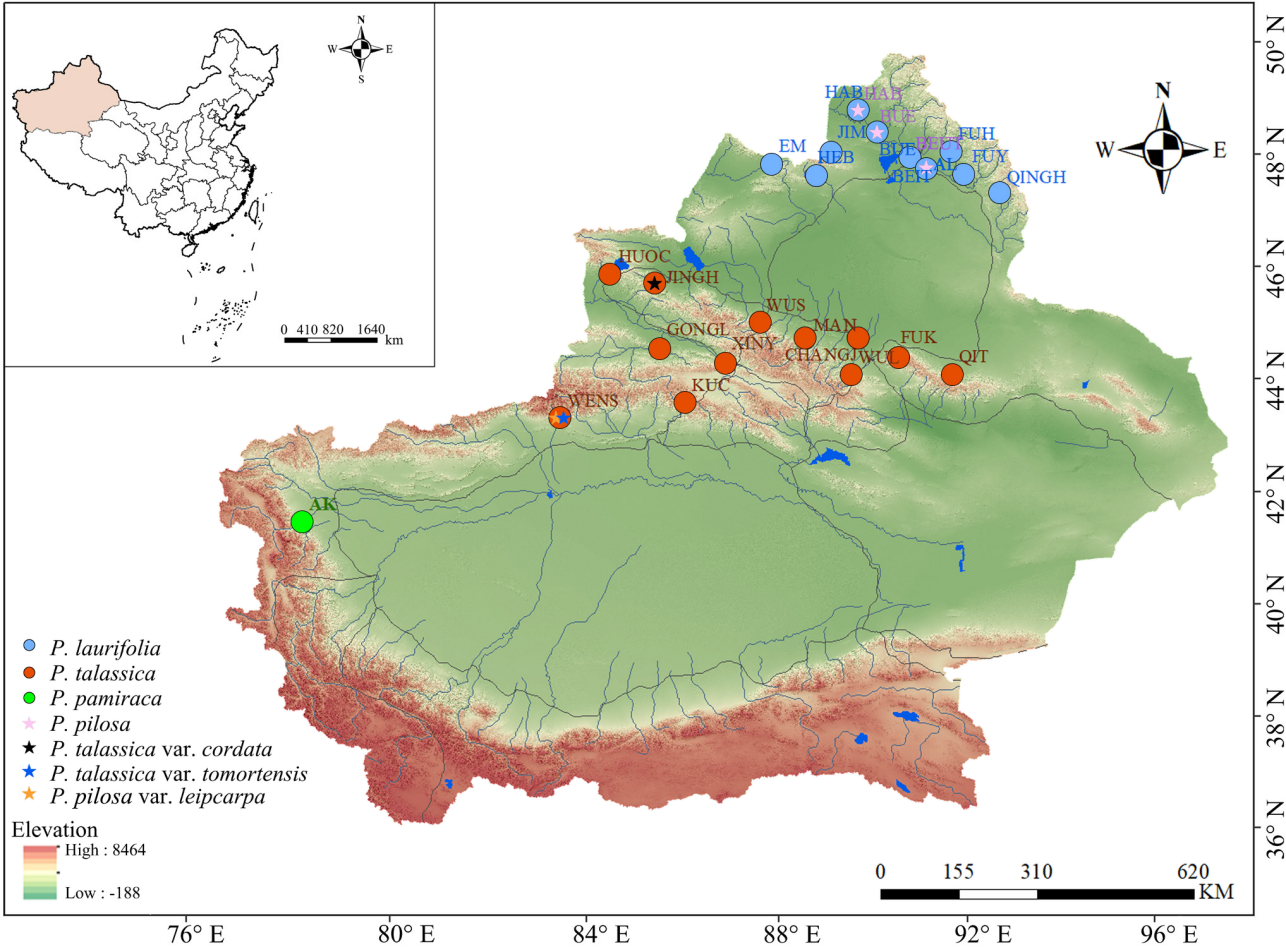
Distribution of sampling points for the *Populus laurifolia* complex species.

Four species and three varieties of *P. laurifolia* complex were examined in this study. Based on available specimen records, collection points were made to cover each species distribution site as much as possible. A total of 337 samples were eventually collected from 29 populations from Xinjiang, China, with the number of individuals sampled per population ranging from a few to approximately 20, depending on the population size. (**Figure 1**, **Supplementary Table S1**). Few samples of *P. pamirica* (five samples) and *P. pilosa* (11 samples) were collected due to their small population sizes in nature. The collected specimens were deposited in the herbarium of the Museum of Beijing Forestry University (BJFC). In addition to field-collected specimens, digital specimens from the Museum of Xinjiang Agricultural University, the National Plant Specimen Resource Center (CVH; https://www.cvh.ac.cn/), the National Specimen Information Infrastructure (NSII; http://www.nsii.org.cn/), JSTOR Global Plants (https://plants.jstor.org), and the Moscow Digital Herbarium (https://plant.depo.msu.ru/) were utilized for morphological analysis.

### 2.2 Morphometric analysis

**Figure 2:**
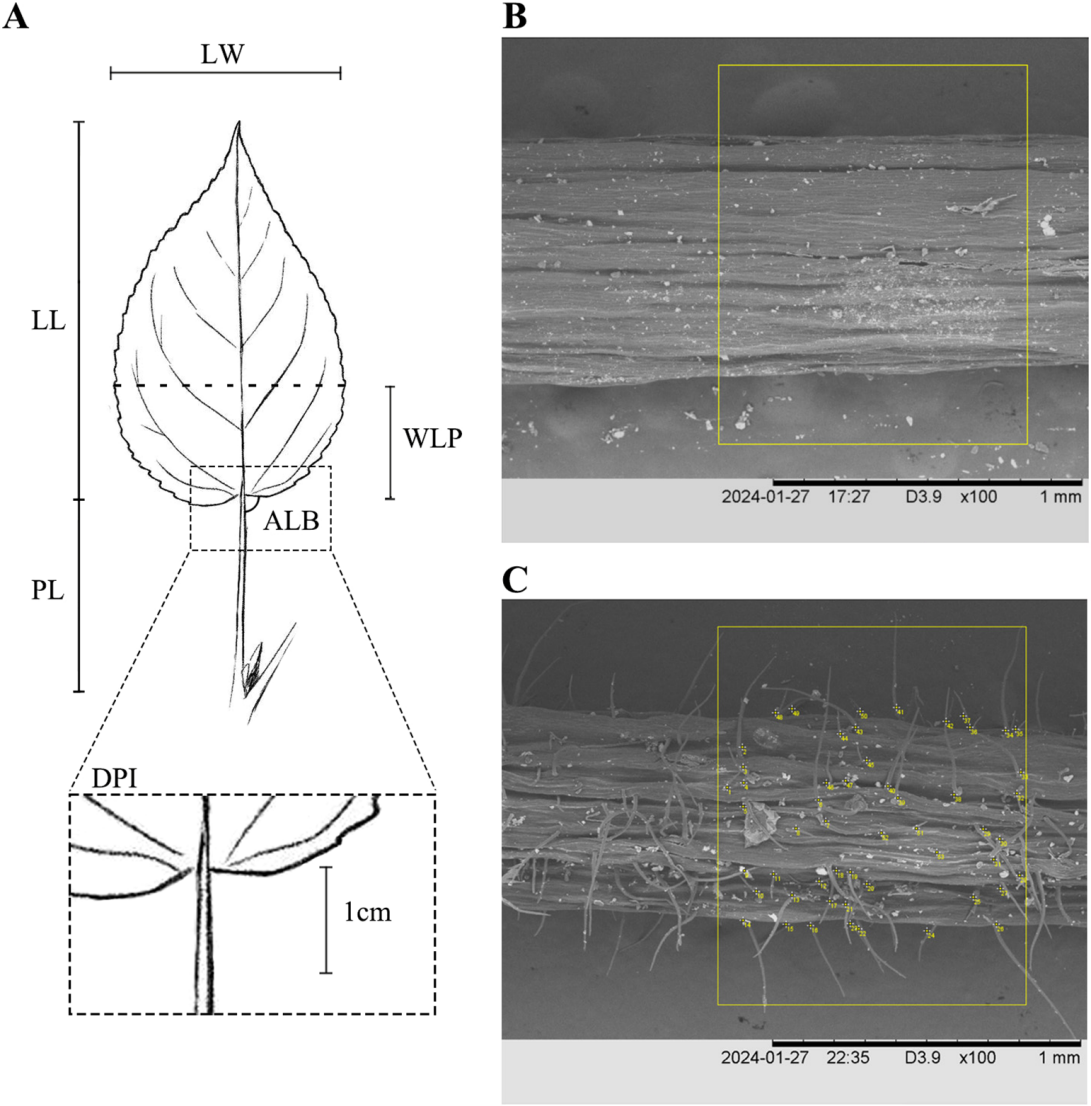
Morphological analyses of *Populus laurifolia* complex species. **(A)** Morphometric measurements in this study: leaf length (LL), leaf width (LW), petiole length (PL), the widest position of the leaf blade (WLP), the base angle of the leaf (ALB), and the density of petiole pubescence (DPP). **(B)** The number of 1-mm^2^ hairs on the petiole of *Populus talassica*. **(C)** The number of 1-mm^2^ hairs on the petiole of *P. laurifolia*.

The morphology of sprout leaves and short-branched leaves, as well as the density of pubescence on petioles and branches, are important characteristics for identifying species in the *P. laurifolia* complex, as supported by *Flora of Xinjiangensis* (Yang, 1993) and our field observations. Eight leaf morphological traits (**Figure 2A**) for both sprout leaves and short-branched leaves of the four species were measured, including leaf length (LL), leaf width (LW), petiole length (PL), the widest position of the leaf blade (WLP), the base angle of the leaf (ALB), leaf width-to-length ratio (LWR), the length from the widest point to the leaf base to the leaf length ratio (WLR), and petiole length-to-leaf length ratio (PLR). Additionally, the density of petiole pubescence (DPP) of short-branched leaves was quantified. The density of pubescence has been designated as a qualitative character in some morphological studies, but the definition of pubescence density is ambiguous and difficult to understand in practice. To better characterize the differences in pubescence density among the species of the *P. laurifolia* complex, petiole hairs were examined using a scanning electron microscope (HITACHI S-4800, Tokyo, Japan). First, a petiole measuring approximately 1 cm in length was extracted from the leaf base and subsequently imaged using a scanning electron microscope at a magnification of ×100, and then the number of pubescence within a 1-mm length of the petiole was quantified utilizing the ImageJ software (Schneider et al., 2012) (**Figure 2**). For the two varieties of *P. talassica*, only the eight leaf morphological traits of short-branched leaves and the DPP were measured.

The above data were processed and analyzed using the Origin 2022 software (Deschenes, 2000). The Kruskal–Wallis tests and Wilcoxon rank sum tests were used to assess the differences in each trait among the four species of the complex. The significance of differences was indicated on box plots. To prevent collinearity in subsequent analyses, highly correlated characteristics were removed using Pearson’s correlation analysis. All statistical analyses were conducted in IBM SPSS Statistics 26, resulting in the retention of three morphological traits. A principal component analysis (PCA) was then performed on these retained traits. To verify the classification of the *P. laurifolia* complex, which is outlined in *Flora of Xinjiangensis* (Yang, 1993) as having heteromorphic leaves for *P. laurifolia* and *P. talassica* and almost homomorphic leaves for *P. pilosa* and *P. pamirica*, PCAs on the morphological traits of sprout leaves and short-branched leaves for each species were conducted.

### 2.3 Phylogenetic analyses

#### 2.3.1 DNA extraction and sequencing

One to six individuals were randomly selected from each population of the *P. laurifolia* complex (**Supplementary Table S2**). Healthy and fresh leaves were collected and immediately dried using silica gel. Genomic DNA was extracted from these silica gel-dried leaves using a modified Cetyltrimethylammonium Bromide (CTAB) method (Doyle, 1987). All sequencing data have been deposited at the National Genomics Data Center (Chen et al., 2021; CNCB-NGDC Members and Partners et al., 2024). All DNA samples were sequenced at Novogene (Beijing, China) using the Illumina NovaSeq 6000 platform (Illumina, San Diego, CA, USA), targeting a 20× coverage for whole-genome paired-end reads. Additionally, to verify the monophyly of the *P. laurifolia* complex, 31 sequences of *Populus* (**Supplementary Table S2**) were downloaded from the National Center for Biotechnology Information (NCBI) database and the Genome Sequence Archive (GSA) in the BIG Data Center for analysis. These sequences included one sample of *P. talassica* and two samples of *P. pamirica* (**Supplementary Table S2**). In addition, four *Populus* samples outside the *P. laurifolia* complex were collected from our own fieldwork for further comparison.

#### 2.3.2 SNP calling

Nuclear variants were identified using GATK tools. The resequencing data were first mapped to the reference genome of *P. koreana* (Sang et al., 2022) using BWA-MEM v.0.7.17-r1188 (Li and Durbin, 2009). Mapped reads were then converted to BAM files, sorted, and filtered using SAMtools v.1.6 (Li et al., 2009), and PCR duplicates were marked using Picard v.2.1.1. Short variants were called using GATK v.4.1.4 (McKenna et al., 2010) with HaplotypeCaller, and the variants from all samples were combined using GATK’s CombineGVCFs. Single-nucleotide polymorphisms (SNPs) were identified using the SelectVariants tool (Danecek et al., 2011). To convert the SNPs into PHYLIP format, Python v.2.7.5 was used, followed by dataset analysis in IQ-TREE v.2.0.3 (Nguyen et al., 2015).

#### 2.3.3 Phylogenetic inference

A maximum likelihood (ML) phylogenetic tree was constructed using IQ-TREE, with the SH-aLRT branch test (1,000 repetitions) specified by the “-alrt” option (Guindon et al., 2010). The most appropriate evolutionary model was selected using ModelFinder (Kalyaanamoorthy et al., 2017). The sliding-window method (Takuno et al., 2012; Zhang et al., 2016; Liu et al., 2017; Chen et al., 2019) was employed to reconstruct the species tree, with concatenated SNPs filtered into 4-kb non-overlapping windows across the genomic alignments. *Populus euphratica* was used as the outgroup.

Additionally, to verify the monophyly of the *P. laurifolia* complex, 31 sequences of *Populus* (**Supplementary Table S2**) were downloaded from the NCBI database and the GSA in the BIG Data Center for analysis. These sequences included one sample of *P. talassica* and two samples of *P. pamirica* (**Supplementary Table S2**). In addition, four *Populus* samples outside the *P. laurifolia* complex were collected from our own fieldwork for further comparison.

#### 2.3.4 Genetic structure analysis

Linkage disequilibrium (LD)-based SNP filtering was conducted using PLINK v.1.9.0 (Purcell et al., 2007). PCA of the filtered *Populus* species was performed using PLINK, and visualizations were created using the R package ggplot2 (Villanueva and Chen, 2019). Population structure was analyzed using ADMIXTURE (Alexander et al., 2009), with the number of clusters (K) set from 1 to 10. Cross-validation was used to determine the optimal K value, with the lowest cross-validation error indicating the most appropriate number of clusters. To evaluate potential hybridization, genetic data from nine *Populus nigra* individuals were downloaded from NCBI, and a genetic structure analysis was performed (**Supplementary Table S2**).

### 2.4 ABBA–BABA analysis

To investigate gene flow from other species into *P. laurifolia* complex, ABBA–BABA statistics (Malinsky et al., 2021) were employed to quantify potential gene exchange. This statistical method assesses deviations from a strictly bifurcated evolutionary history using genome-scale SNP data, thereby testing for gene penetrance (Martin et al., 2015; Malinsky et al., 2021). Patterson’s D-statistic (Durand et al., 2011) was calculated using the Dtrios program from Dsuite (Malinsky et al., 2021). In this study, for the ordered alignment {[(P1, P2), P3], O}, the ABBA site pattern represents the shared derived alleles of P2 and P3, while the BABA site pattern corresponds to the shared derived alleles of P1 and P3. Under the null hypothesis of incomplete lineage sorting (ILS), the number of ABBA sites and BABA sites is expected to be equivalent (D = 0). A significant deviation of D from 0 suggests alternative evolutionary events, particularly indicating potential gene exchange between P3 and either P1 or P2 (Durand et al., 2011). If the absolute value of the Z-score exceeds 3, it is considered statistically significant, indicating a strong deviation from the expected distribution (Busing et al., 1999). Subsequently, F-branch analysis further quantifies the relative strength of gene flow and maps the results onto different branches of the phylogenetic tree to infer the direction and contribution of gene flow. Gene flow intensity between each species and the phylogenetic branch was measured using the F4 ratio, and a heatmap was created for visualization in the Origin 2022 software (Deschenes, 2000).

### 2.5 Niche differentiation analysis

Previously collected specimens from the CVH were re-identified based on key traits, and due to the distribution overlap between *P. pilosa* and *P. laurifolia*, they were combined into a single phenotypic cluster. A total of 212 locations were identified across three clusters, which were used to construct their respective distributional niches. To analyze niche similarities and differences among these phenotypic clusters, 19 bioclimatic variables were first downloaded from the WorldClim database (Hijmans et al., 2005). After missing values were removed, the species occurrence data were combined with background environmental data for a PCA using the dudi.pca function from the ade4 package, reducing the dimensionality of the environmental space. The resulting PCA scores for the three clusters were then used to create dynamic climatic grids via the ecospat.grid.clim.dyn function, representing the species’ niches in environmental space. Subsequently, niche similarities and differences between the clusters were calculated using Schoener’s D and the standardized Hellinger distance (I) in ENMTools 1.3 (Warren et al., 2019). To further assess niche equivalency and similarity, 100 replicates of both tests were performed using the equivalency and similarity functions.

## 3 Results

### 3.1 Phylogenetic evidence for three clades within the *P. laurifolia* complex

**Figure 3:**
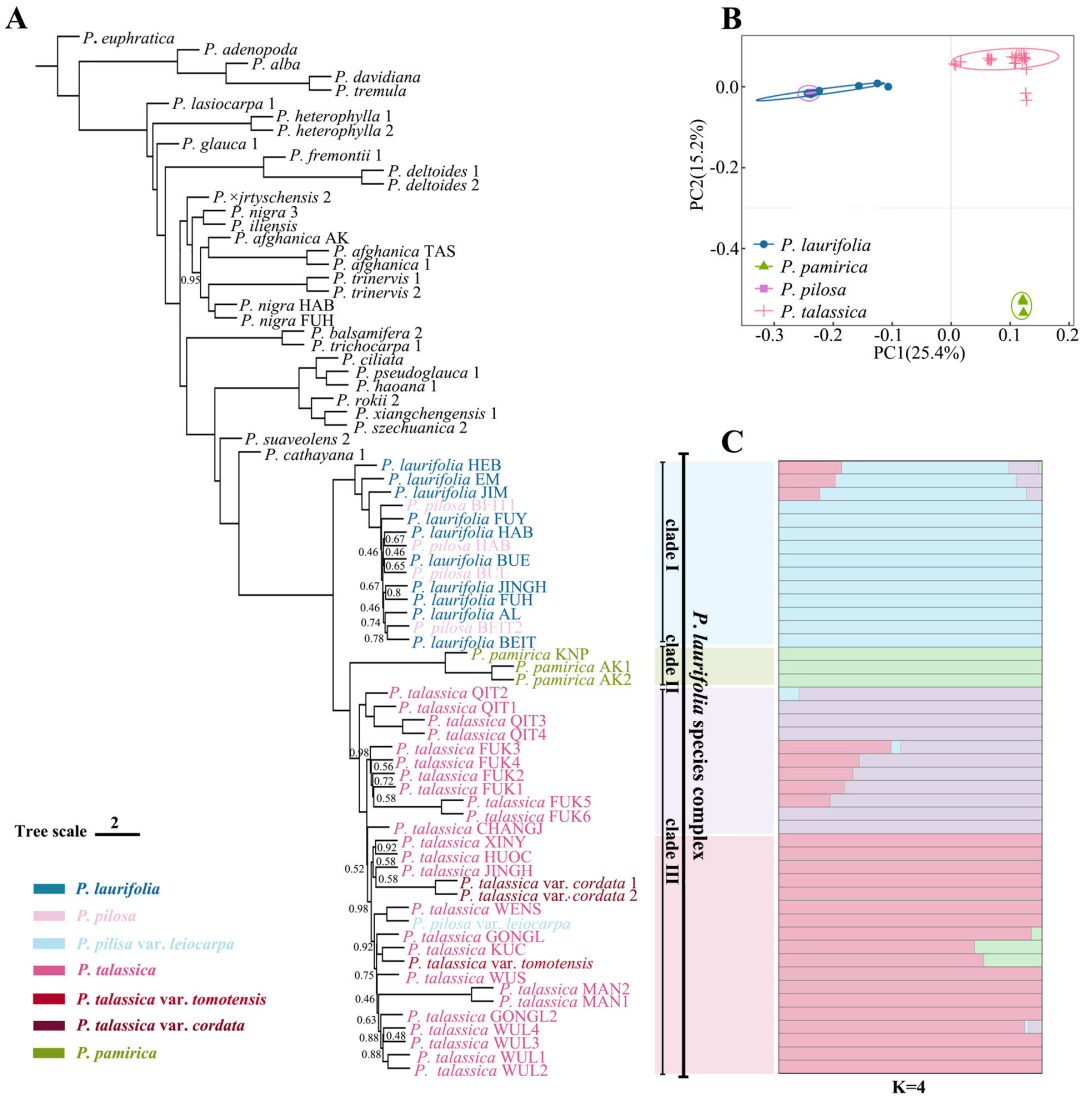
Phylogenetic analyses. **(A)** Phylogeny estimated with alternative quartet topologies (-t 8) in ASTRAL based on 4-kb non-overlapping windows. Unless otherwise indicated, all nodes had 1.0 posterior probability. **(B)** Principal component analysis (PCA), based on genetic distance using SNP data. **(C)** Population structure analysis for four species of *Populus laurifolia* species complex. Each colored bar represents one individual, and colored segments represent proportions of ancestral components. The order of individuals corresponds to their order in the phylogenetic tree in panel **(A)**.

Phylogenetic analyses (**Figure 3A**) based on the ASTAL method showed that the *P. laurifolia* complex formed a well-supported clade (posterior probability = 100), which consisted of three subclades: 1) *P. laurifolia* and *P. pilosa* (Clade I), 2) *P. pamirica* (Clade II), and 3) *P. talassica* and its varieties, along with *P. pilosa* var. *leiocarpa* (Clade III). The *P. talassica* clade was the sister group to the *P. pamirica* clade (PP = 100), and both clades were monophyletic. *Populus laurifolia* and *Populus pilosa* together formed a monophyletic clade, which was a sister to the *P. pamirica* + *P. talassica* clade. The same topology was also recovered by a concatenated tree based on 566,375 nuclear SNPs (**Supplementary Figure S2**). PCA of the SNP data produced consistent results, with the individuals of the *P. laurifolia* complex forming three distinct clusters: *P. pilosa* and *P. laurifolia* clustered together, while *P. talassica* and *P. pamirica* formed separate clusters (**Figure 3B**). The optimal K value for the genetic admixture analysis of the *P. laurifolia* complex was determined to be 4 (**Figure 3C**). The populations of *P. laurifolia* and *P. pilosa* contained the same genetic clusters, indicating minimal genetic differentiation between the two species. *Populus laurifolia* populations from EM, HEB, and JIM also showed genetic components of *P. talassica*, suggesting some gene flow between these populations and *P. talassica* in the Tianshan region. Samples of *P. pamirica* from China and Pakistan were assigned to a single genetic cluster, indicating a distinct and specialized genetic background. For *P. talassica*, two genetic lineages were observed: 1) populations from QIT, CHANGJ, and FUK and 2) populations from other regions. However, this genetic differentiation was not supported by the phylogenetic tree.

### 3.2 ABBA–BABA analysis reveals limited gene flow

**Figure 4:**
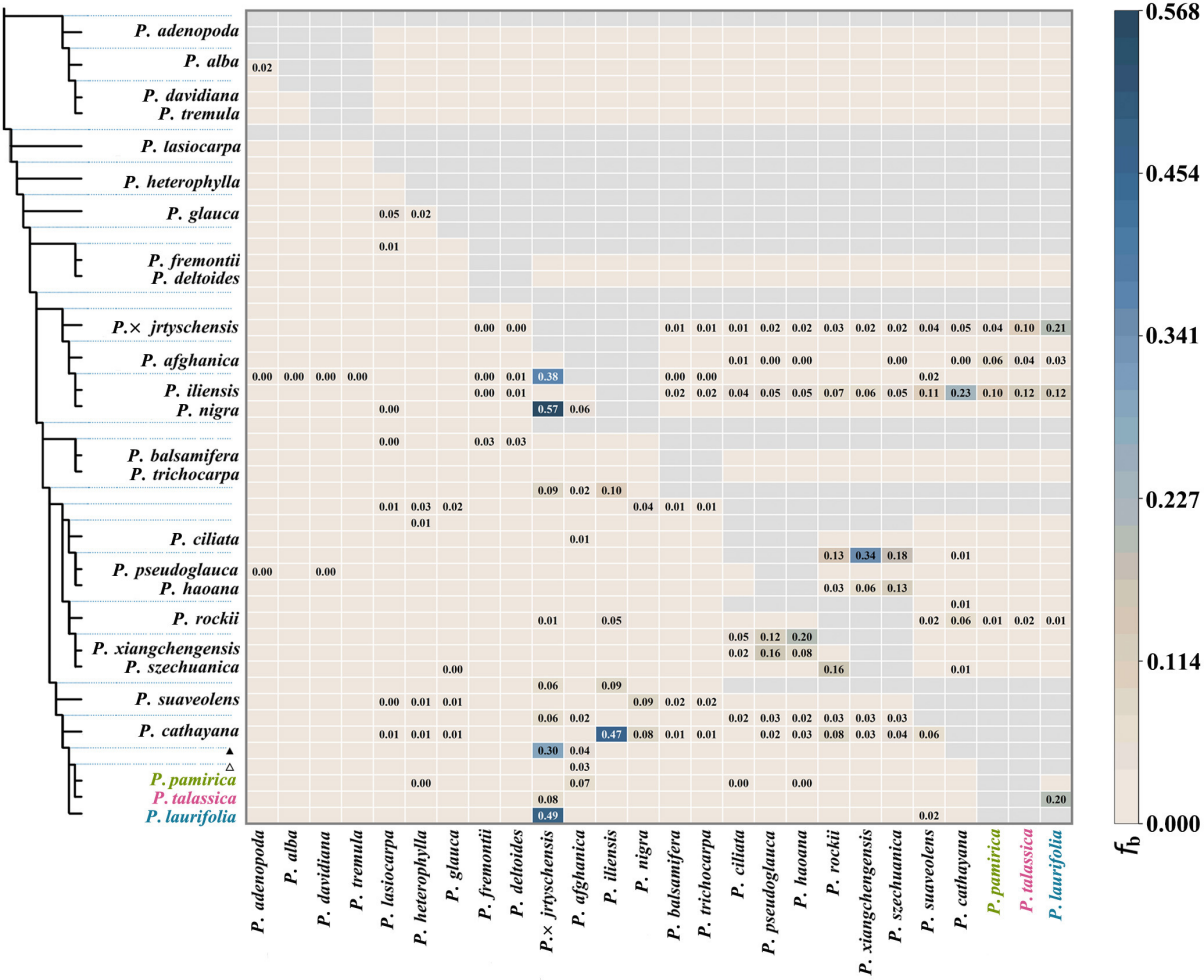
ABBA–BABA statistics results measuring gene flow among 26 species. Gray areas represent no gene flow, and values equal to 0.000 are not labeled. The left panel shows the species tree estimated in ASTRAL using 4-kb non-overlapping windows. Black triangles indicate gene flow between the ancestor of *Populus laurifolia* complex and other species, while hollow triangles show gene flow between ancestor of *Populus pamirica* and *Populus talassica* with other species.

Based on the phylogenetic tree topology, we divided the *P. laurifolia* complex into three monophyletic taxa: *P. laurifolia* (including *P. pilosa*), *P. pamirica*, and *P. talassica*. To further investigate gene flow within this complex, we used *P. euphratica* as an outgroup and applied the ABBA–BABA method (**Figure 4**). The result showed that significant gene exchange was absent within this complex. Although some gene flow occurred between *P. talassica* and *P. laurifolia*, the overall level remained low. In contrast, gene exchange between *Populus × jrtyschensis* and *P. laurifolia* occurred more frequently, with active gene flow between *P. × jrtyschensis* and the broader *P. laurifolia* complex. This increased gene exchange may be attributed to *P. laurifolia* serving as a parental species of *P. × jrtyschensis* (Jiang et al., 2016).

### 3.3 Morphological analyses

#### 3.3.1 Morphological traits of short-branched leaves

**Figure 5:**
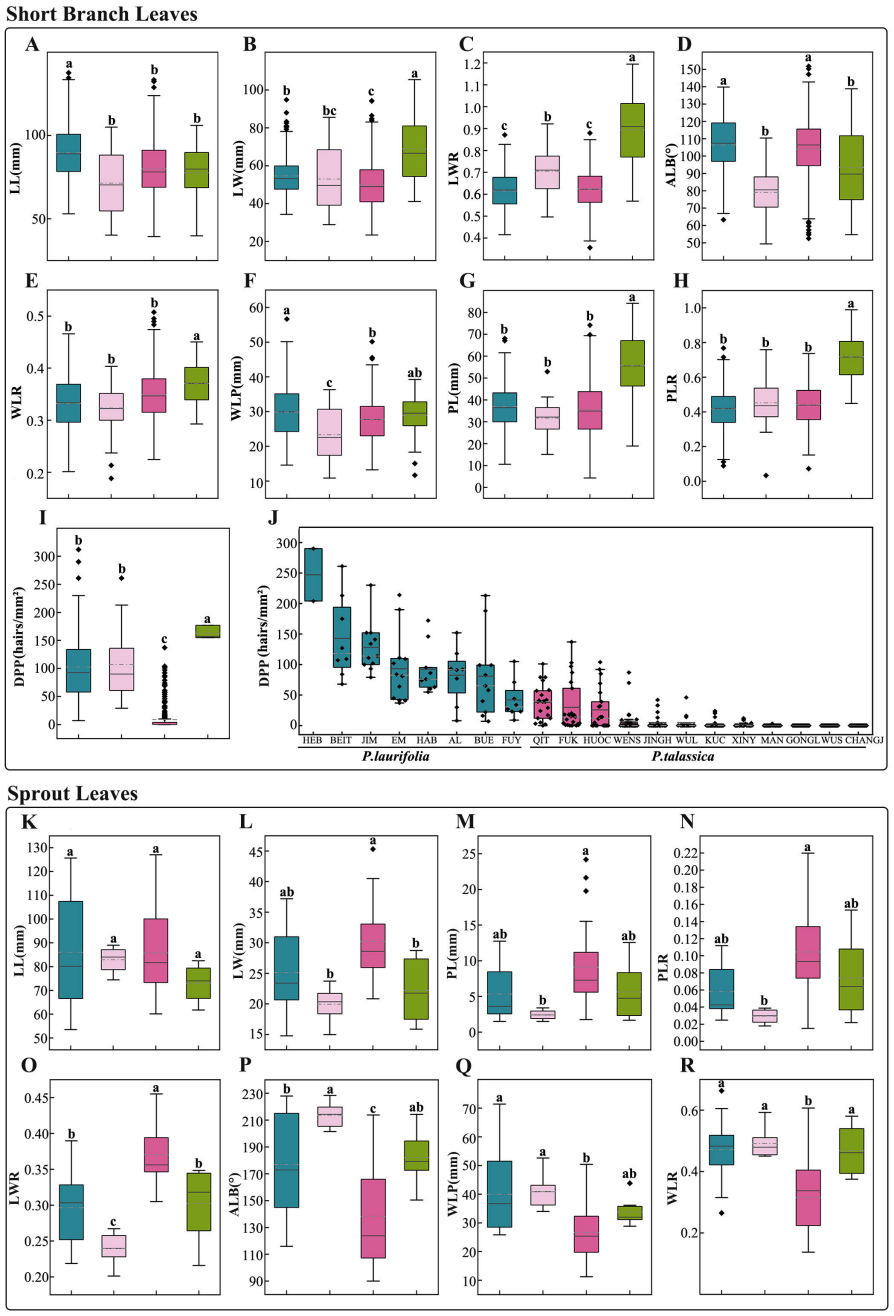
Morphological comparison of short-branched leaves **(A–J)** and sprout leaves **(K–R)** among species of the *Populus laurifolia* complex. **(A, K)** Leaf length (LL). **(B, L)** Leaf width (LW). **(C, O)** Leaf width-to-length ratio (LWR). **(D, P)** Leaf base angle (ALB). **(E, R)** Ratio of the distance from the widest point to the leaf base to the leaf length (WLR). **(F, Q)** Position of the widest point of the leaf blade (WLP). **(G, M)** Petiole length (PL). **(H, N)** Petiole length-to-leaf length ratio (PLR). **(I)** Density of petiole pubescence (DPP) among four species. **(J)** DPP of short-branched leaves among populations of *P. laurifolia* and *Populus talassica*. Boxplot colors represent species: blue for *P. laurifolia*, pink for *Populus pilosa*, red for *P. talassica*, and green for *Populus pamirica*. The gray line indicates the mean, and the light gray dashed line indicates the median. Different lowercase letters above columns show significant trait differences (p < 0.05); shared letters denote none.

The short-branched leaves of the *P. laurifolia* complex are predominantly ovate or long-ovate with finely serrated edges. A quantitative analysis of eight morphological traits revealed distinct differences among species (**Figure 5**; **Supplementary Table S3**). Although the leaves of *P. laurifolia*, *P. talassica*, and *P. pilosa* shared similar shapes (**Figure 5**), *P. pilosa* was characterized by predominantly cordate leaf bases (**Figure 5D**), while both *P. laurifolia* and *P. talassica* exhibited ovate bases (**Figure 5D**). This distinction in leaf base shape is a primary criterion for differentiating *P. pilosa* from the other two species. In contrast, *P. pamirica* showed significant morphological differences compared to the other species in the complex. Notably, *P. pamirica* had wider leaves (**Figure 5B**) and a higher LWR (**Figure 5C**), along with a greater petiole length (**Figure 5G**) and PLR (**Figure 5H**) compared to *P. laurifolia*, *P. talassica*, and *P. pilosa*. Further analysis using Pearson’s correlation indicated that LL, LW, WLP, WLR, and ALB did not significantly contribute to species differentiation and were excluded from subsequent analyses. PCA, based on the remaining variables (PL, PLR, and LWR), suggested that while differentiating the four species is challenging, *P. pamirica* remained relatively distinct with minimal overlap with the other species (**Supplementary Figure S1B**).

Petiole pubescence density is a key trait distinguishing the *P. laurifolia* complex from other species. Among the complex, *P. pamirica* exhibited the highest petiole hair density, while no significant difference in hair density was observed between *P. laurifolia* and *P. pilosa*, while *P. talassica* had the lowest density, with only 9.23 hairs/mm^2^ on average (**Figure 5I**; **Supplementary Table S3**). Geographic variation in *P. talassica* revealed significant differences: populations from Gongliu, Wusu, and Changji had glabrous petioles, while those from Qitai, Fukang, and Huocheng were tomentose (**Figure 5J**). These findings were consistent with the description in the *Flora of Xinjiangensis* (Yang, 1993), which classified the densely pubescent petioles in Wensu County as *P. talassica* var. *tomortensis*. However, our data suggested that petiole pubescence in *P. talassica* exhibited significant variation, indicating that it may not reliably reflect varietal differences. While the short branchlet leaves of *P. talassica* and *P. laurifolia* were morphologically similar, populations of *P. laurifolia* generally possessed higher petiole hair density, ranging from 7 to 312 hairs/mm^2^, in contrast to the predominantly glabrous *P. talassica* populations. This key difference underscored the morphological distinction between the two species.

#### 3.3.2 Morphological traits of sprout leaves

The sprout leaves of species within the *P. laurifolia* complex, including *P. laurifolia*, *P. pilosa*, *P. talassica*, and *P. pamirica*, were primarily obtained from online digitized herbarium specimens. The results (**Figure 5**; **Supplementary Table S4**) revealed that *P. laurifolia* and *P. pamirica* exhibited a high degree of similarity in sprout leaf morphology, while *P. laurifolia* and *P. pilosa* also showed similar traits, although *P. pilosa* was characterized by narrower sprout leaves (**Figure 5O**). In contrast, *P. talassica* showed significantly higher values of LWR (**Figure 3**O) and lower values of WLP and WLR (**Figures 5Q, R**) compared to the other species. Additionally, the leaf base angle of *P. talassica* was more oval-shaped compared to that of the other species (**Figure 5P**).

#### 3.3.3 Morphological variation in *P. laurifolia* complex

**Figure 6:**
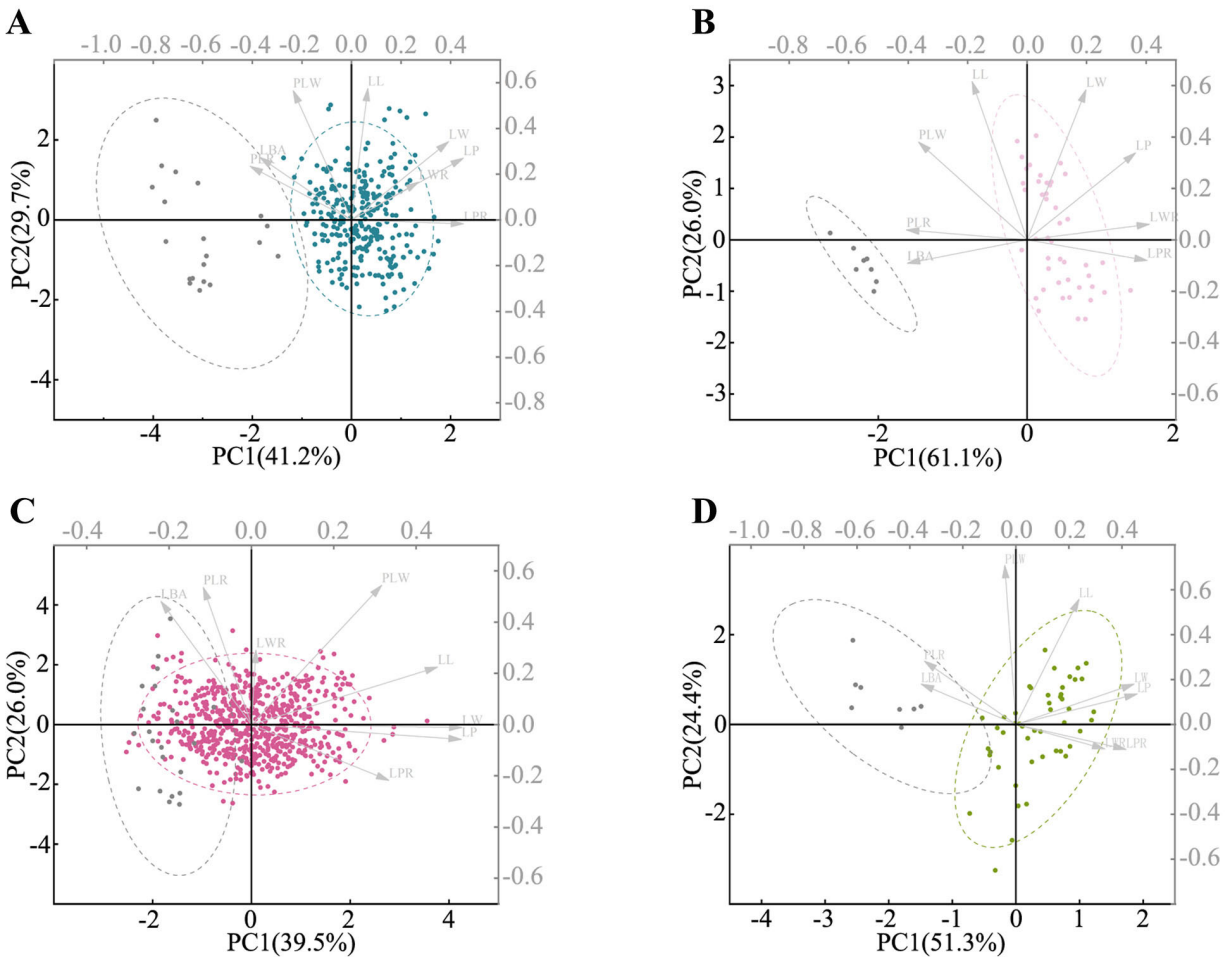
Principal component analysis (PCA) based on morphometric data. **(A)** PCA plot comparing the short-branched leaves (in blue) and sprout leaves (in gray) of *Populus laurifolia*. **(B)** PCA plot of the short-branched leaves (in light pink) and sprout leaves (in gray) of *Populus pilosa.* **(C)** PCA plot of the short-branched leaves (in red) and sprout leaves (in gray) of *Populus talassica*. **(D)** PCA plot of the short-branched leaves (in olive green) and sprout leaves (in gray) of *Populus pamirica*.

The PCA results demonstrated significant morphological differentiation between short-branched leaves and sprout leaves within the *P. laurifolia* complex, except for *P. talassica*, which showed no significant differences (**Figure 6**). In *P. laurifolia* (**Figure 6A**), short-branched leaves (in blue) clustered distinctly from sprout leaves (in gray), with the former exhibiting lanceolate shapes compared to elliptic, ovate, or oblong-ovate shapes of the latter. Similarly, *P. pilosa* (**Figure 6B**) showed a clear separation between short-branched leaves (in light pink) and sprout leaves, underscoring their distinct morphological characteristics. The PCA for *P. talassica* (**Figure 6C**) revealed that short-branched leaves (in red) were broader and more ovate, whereas the sprout leaves were narrower and lanceolate. A similar trend was observed in *P. pamirica* (**Figure 6D**), highlighting substantial morphological variation between the two leaf types.

#### 3.3.4 Other morphological traits

The species within the *P. laurifolia* complex shared several similarities, including comparable inflorescences (**Supplementary Figures S3G, N**) and ovoid capsules that were three- or four-valved (**Supplementary Figures S4C, S3F, M**). However, they also exhibited distinct differences. For the species we examined, the capsules of *P. laurifolia* and *P. pilosa* were densely covered with woolly hairs (**Supplementary Figures S4C, S3C**), whereas *P. talassica* had capsules with few to no hairs (**Supplementary Figure S3M**). The branchlets of *P. laurifolia* and *P. pamirica* were hairy, while those of *P. talassica* were nearly hairless (**Supplementary Figure S3**). Additionally, *P. laurifolia* had yellowish, distinctly angled, and tomentose sprouts (**Figure 5E**, **Figures 6C**), whereas *P. talassica* exhibited russet or gray suckers and glabrous, slightly angled sprouts (**Supplementary Figures S6J, K, L**).

### 3.4 Niche differentiation analysis

During our field survey, we identified several specimens with incorrect species identifications at various recording sites, where the actual poplar species present differed from those recorded. For instance, in the Tianchi Lake Scenic Area of Fukang County, individuals previously identified as *P. laurifolia* or *P. pilosa* were found to be *P. talassica*. Our survey revealed no distribution overlap among *P. talassica*, *P. laurifolia*, and *P. pamirica* (**Figure 1**). We employed Schoener’s D and Warren’s I indices to assess niche differentiation between these species. The results (**Table 1**) indicated very low ecological overlap between *P. pamirica* and both *P. talassica* and *P. laurifolia*, signifying a high degree of niche differentiation for *P. pamirica*. Conversely, the overlap indices for *P. laurifolia* and *P. talassica* were relatively high, suggesting minimal niche differentiation between these two species. Despite the observed niche overlap, the field distributions of these species do not intersect, implying that geographic isolation may be a primary factor driving their differentiation.

**Table 1:** Niche divergence comparisons among three species using Schoener’s D and Warren’s I.

Species		*Populus laurifolia*	*Populus talassica*	*Populus pamirica*
		Schoener’s D		
*P. laurifolia*	Warren’s I		0.562	0.019
*P. talassica*		0.747		0.170
*P. pamirica*		0.040	0.305	

The top half is Schoener’s D result, and the bottom half is Warren’s I result.

## 4 Discussion

### 4.1 Species delimitation in *P. laurifolia* complex

Establishing species boundaries among closely related species with a history of hybridization poses significant challenges. However, statistical analyses incorporating multiple lines of evidence suggest that an integrative approach is more effective (Liu, 2015, 2016). Traditionally, the *P. laurifolia* complex was considered to include four species—*P. laurifolia*, *P. pilosa*, *P. talassica*, and *P. pamirica*—distinguished primarily by leaf shape and petiole pubescence (Yang, 1993; Fang et al., 1999). In contrast, Skvortsov (2009, 2010) proposed that *P. pamirica*, *P. talassica*, and *P. pilosa* should be classified as a single species, with *P. macrocarpa* as the valid name. However, our study supports merging *P. pilosa* with *P. laurifolia* while recognizing *P. talassica* and *P. pamirica* as distinct species.

Phylogenetic analysis based on nuclear SNP data reveals that the *P. laurifolia* complex forms three well-supported clades (PP = 100), with *P. talassica* and *P. pamirica* being more closely related than *P. laurifolia* (including *P. pilosa*) (**Figure 3A**). This clustering is further supported by the PCA result (**Figure 3B**). These molecular findings suggest that *P. pilosa* and *P. laurifolia* may not be distinct at the species level. Morphological analysis supports this hypothesis. Our morphological analysis (**Figure 5**) shows that the short-branched and sprout leaves of *P. laurifolia* and *P. pilosa* are very similar, with only minor differences, such as the cordate leaf base of *P. pilosa*’s short-branched leaves and slightly narrower sprout leaves. These differences are heavily influenced by environmental factors and are not stable (Gong et al., 2023; Zhao et al., 2023). Furthermore, *P. pilosa* shares the same habitat as *P. laurifolia*, lacking an independent distribution area (**Figure 1**). Moreover, the original descriptions of *P. pilosa* lacked detailed information on sprout leaves. Together, this evidence supports the hypothesis that *P. pilosa* is a morphological variant of *P. laurifolia*.

In contrast, *P. talassica* and *Populus pamirica* exhibit distinct morphological and molecular features. *Populus pamirica* can be distinguished by the leaf shape of its short-branched leaves, which are nearly equal in length and width, its longer petioles, and the denser pubescence on the branchlets and petioles (**Figure 5**; **Supplementary Figures S3, S4**). *Populus talassica* was often misidentified as *Populus laurifolia* in the field, but our morphological analysis shows significant differences, particularly in pubescence (**Figures 5I, J**). Although both species have similar short branchlet leaves, *P. talassica* exhibited less pubescence, with some nearly glabrous specimens. Specimens from Fukang and Qitai with dense pubescence were misidentified, but *P. talassica* consistently shows lower petiole pubescence than *P. laurifolia*. Additional differences included less lanceolate sprout leaves, non-angled sprout morphology, nearly glabrous capsules, and distinct geographical distributions (**Figures 1**, **5**, **6**; **Supplementary Figure S3**).

To accurately classify the *Populus* species, especially within the *P. laurifolia* complex, an integrative approach is essential. This approach should incorporate multiple morphological traits from various plant parts. The combined morphological, genetic, and ecological data strongly support the re-evaluation of the *P. laurifolia* complex. The evidence indicates that *P. laurifolia* (including *P. pilosa*), *P. talassica*, and *P. pamirica* should be treated as three independent species, reflecting their distinct evolutionary trajectories and ecological adaptations. Future taxonomic revisions should consider these findings to enhance the understanding and classification of this complex.

The *P. laurifolia* complex in *Flora of Xinjiangensis* and *FRPS* included four varieties: *P. talassica* var. *cordata*, *P. talassica* var. *tomortensis*, *P. pamirica* var. *akqiensis*, and *P. pilosa* var. *leiocarpa*. In this study, we examined specimens of *P. talassica* var. *cordata* and *P. talassica* var. *tomortensis* collected from their type localities (Jinghe and Wensu, Xinjiang) and compared them with *P. talassica* populations (**Supplementary Figure S1C**). The distinguishing characteristic of *P. talassica* var. *cordata* was its cordate leaf base; however, *P. talassica* exhibited variable leaf bases (cuneate, truncate, or cordate) even within the same tree, complicating its differentiation. Similarly, *P. talassica* var. *tomortensis* was described as having broadly ovate leaves and densely tomentose short branches, petioles, veins, and infructescence axes. We observed that these traits also appeared in other *P. talassica* populations (e.g., Fukang and Qitai), suggesting that these features were inconsistent and not supported by phylogenetic analysis, casting doubt on the validity of these varieties. We obtained specimens of *P. pilosa* var. *leiocarpa* from its type locality (Tomur Peak, Wensu County) and also collected *P. talassica* var. *tomortensis* in the same area (**Supplementary Figures S5, S6**). The latter also exhibited densely tomentose infructescence axes and glabrous capsules, raising the possibility that *P. pilosa* var. *leiocarpa* may represent a misidentification or simply a variant of *P. talassica*. Phylogenetic analysis based on nuclear SNP data supported this hypothesis.

Based on our morphological and phylogenetic analysis, we conclude that the recognition of *P. talassica* var. *cordata*, *P. talassica* var. *tomortensis*, and *P. pilosa* var. *leiocarpa* as distinct varieties is unwarranted. These varieties should be subsumed under *P. talassica* at the species level.

### 4.2 Geographic isolation and niche differentiation

The *P. laurifolia* species complex, which includes *P. pamirica*, *P. talassica*, and *P. laurifolia* (a division based on the results of previous species boundary discussions), exhibits varying degrees of geographic isolation and ecological differentiation (**Table 1**, **Figure 1**). These three species showed distinct clusters with complete or partial geographical isolation, suggesting that geographic barriers played a significant role in maintaining their separation. *Populus laurifolia* and *Populus talassica* typically thrived in riparian environments, favoring moist habitats along water bodies due to their reliance on sufficient water for growth and survival. In the Junggar Basin, geographic isolation likely drove their speciation, as the region’s arid conditions served as a natural barrier (Carroll et al., 1990; Chen et al., 2002). Despite this, substantial niche differentiation did not occur (Wang et al., 2006). The results of Schoener’s D and Warren’s I indices revealed a relatively high overlap between the ecological niches of *P. talassica* and *P. laurifolia* (**Table 1**). However, the absence of current field distribution overlap suggests that geographic isolation, rather than ecological differentiation, has been the primary driver of their divergence (Anacker and Strauss, 2014). This pattern aligns with the classic model of allopatric speciation, where physical barriers facilitate the formation of distinct species without immediate ecological divergence.

Differently, *P. pamirica* has undergone significant niche differentiation compared to *P. laurifolia* and *Populus talassica* (**Table 1**). It grows at higher elevations and is adapted to colder environments, indicating the evolution of specialized ecological adaptations influenced by its unique geographic and environmental conditions in the Pamir region (Metrak et al., 2015; Mętrak et al., 2017, 2023). Its populations are confined to distinct ecological niches in the Pamir region, as evidenced by low niche overlap indices.

### 4.3 Hybridization and genetic admixture

Natural hybridization and genetic introgression play crucial roles in biological evolution, particularly in influencing species diversity (Harland, 1950; Arnold, 1992; Gompert and Buerkle, 2016). Within the *Populus* genus, extensive gene flow occurs, and several hybrid zones have been confirmed (Lexer et al., 2010; Jiang et al., 2016). However, the *P. laurifolia* complex presents a contrasting pattern, where hybridization and gene flow are more restricted compared to other *Populus* species. Our ABBA–BABA analysis (**Figure 4**) reveals that gene flow within the *P. laurifolia* complex is limited, indicating that species have not undergone significant genetic backflow after divergence. In contrast, hybridization between *P. laurifolia* and *P. nigra* produced *P. × jrtyschensis* (**Figure 4**), with populations predominantly consisting of F1 hybrids, forming a typical hybrid zone (Jiang et al., 2016). This indicates more extensive gene flow between *P. laurifolia* and *P. nigra*, possibly due to the role of *P. laurifolia* as a hybrid parent of *P. × jrtyschensis*. In this study, our genetic structure analysis of *P. laurifolia*, *P. × jrtyschensis*, and *P. nigra* (**Supplementary Figure S1A**) showed that K = 2 was the best fit. The collected *P. laurifolia* samples were genetically pure and showed no evidence of hybridization with *P. nigra*, ensuring the purity of the species within the *P. laurifolia* complex in this study. The limited gene flow within the *P. laurifolia* complex reflects a more stable evolutionary trajectory, where geographic isolation and reproductive barriers play a significant role in maintaining species boundaries. By contrast, the Erqis River Valley, known for its high *Populus* diversity in Xinjiang, may have facilitated cross-species hybridization involving *P. laurifolia* (Jiang et al., 2016). Meanwhile, *P. pamirica* remains relatively independent genetically (**Figure 4**), with lower levels of gene flow likely due to ecological or reproductive isolation, which allows it to maintain its distinct genetic characteristics. Furthermore, genetic structure analysis showed that K=4 was the optimal model (**Figure 5C**), with a few individuals in each cluster exhibiting mixed genetic components, indicative of minor introgression between species. Additionally, *P. talassica* was divided into two genetic components (**Figure 5C**), suggesting that despite its relatively uniform morphology, substantial genetic differentiation exists within the species. However, this genetic differentiation has not yet resulted in further morphological or phylogenetic divergence and may be linked to ecological adaptation. Further research is needed to understand the causes of this genetic split and its evolutionary significance.

## 5 Conclusion

In this study, we conducted extensive sampling of the *P. laurifolia* complex to clarify species boundaries through morphological and genetic analyses. The results supported the classification of this complex into three distinct species: *Populus laurifolia*, *Populus talassica*, and *Populus pamirica*. *Populus pilosa* is considered a synonym of *P. laurifolia* due to the absence of significant morphological or genetic divergence. Although some varieties within this complex exhibit subtle morphological differences, these variations do not form monophyletic groups on the phylogenetic tree. Therefore, this study does not support recognizing infraspecific taxa (varieties) within the complex.

Morphological comparisons revealed that *P. laurifolia* and *P. pilosa* shared very similar traits, with only minor differences in leaf morphology that were insufficient to justify their separation at the species level. In contrast, *P. talassica* is distinct, exhibiting differences in leaf shape, pubescence, and sprouts. Additionally, its geographic distribution did not overlap with that of *Populus laurifolia* or *Populus pamirica*. *Populus pamirica* was clearly distinguishable by its short branchlet leaf morphology and geographic isolation in the Pamir region. Phylogenetic analyses were consistent with these findings and supported the classification of the complex into three distinct species, with *P. talassica* and *P. pamirica* forming a sister group. Our ABBA hybridization analysis revealed limited gene flow within the *P. laurifolia* complex, with ecological niche differentiation also being minimal. In contrast, geographic isolation appeared to play a more significant role in speciation within this complex. Future research should explore how geographic isolation interacts with ecological factors to drive speciation within the *Populus* genus. This study emphasized the importance of a multifaceted approach—incorporating morphological, genetic, and ecological data—when clarifying species boundaries. By clarifying the taxonomic structure of the *P. laurifolia* complex, our findings provided valuable insights for future classification and the evolutionary understanding of the *Populus* species.

## Data Availability

The data presented in the study are deposited in the Genome Sequence Archive in National Genomics Data Center (https://ngdc.cncb.ac.cn/gsa/), accession number: CRA018863 (GSA).
