# Supplementary material for: Species delimitation in the Populus laurifolia complex (Salicaceae) based on phylogenetic and morphometric evidence
Source: Front Plant Sci. 2025 Feb 6;16:1518122. doi: 10.3389/fpls.2025.1518122 (PMC11839596; doi:10.3389/fpls.2025.1518122)
Supplement: Supplementary file 1 [file Table1.docx]

**Table S1.** Collection sites and geographic information for the 29 populations of the taxa of the *Populus laurifolia* complex in Xinjiang.

| **Taxon/Population**  **number** | **Locality** | **Longitude** | **Latitude** | **Elevation** |
| --- | --- | --- | --- | --- |
| ***P. laurifolia*** |  |  |  |  |
| JIM | Jimunai(Xinjiang) | 85.77441 | 47.29541 | 1367 |
| EM | Emin(Xinjiang) | 84.35465 | 46.86600 | 550 |
| HEB | Hebukesaier(Xinjiang) | 85.39003 | 46.80146 | 1354 |
| HAB | Habahe(Xinjiang) | 86.34775 | 48.07620 | 526 |
| BUE | Buerjin(Xinjiang) | 86.83520 | 47.71556 | 471 |
| FUY | Fuyun(Xinjiang) | 89.31720 | 47.21570 | 1039 |
| AL | Aletai(Xinjiang) | 88.30049 | 47.26416 | 673 |
| BEIT | Beitun(Xinjiang) | 87.89171 | 47.34029 | 497 |
| QINGH | Qinghe(Xinjiang) | 90.27941 | 46.94175 | 1560 |
| FUH | Fuhai(Xinjiang) | 88.88083 | 47.58500 | 914 |
| ***P. talassica*** |  |  |  |  |
| JINGH | Jinghe(Xinjiang) | 82.17383 | 44.44352 | 640 |
| HUOC | Huocheng(Xinjiang) | 81.01991 | 44.39975 | 1276 |
| GONGL | Gongliu(Xinjiang) | 82.51538 | 43.33744 | 875 |
| XINY | Xinyuan(Xinjiang) | 84.11673 | 43.30884 | 1431 |
| KUC | Kuche(Xinjiang) | 83.37410 | 42.46407 | 2481 |
| WENS | Wensu(Xinjiang) | 80.57537 | 41.76357 | 2355 |
| WUS | Wusu(Xinjiang) | 84.77127 | 44.08649 | 1512 |
| WUL | Wulumuqi(Xinjiang) | 87.16989 | 43.42830 | 2035 |
| FUK | Fukang(Xinjiang) | 88.12756 | 43.88789 | 1917 |
| QIT | Qitai(Xinjiang) | 89.59880 | 43.66531 | 1588 |
| CHANGJ | Changji(Xinjiang) | 87.09536 | 44.08472 | 567 |
| MAN | Manasi(Xinjiang) | 85.99448 | 43.87640 | 1403 |
| var. *cordata* |  |  |  |  |
| JINGH | Jinghe(Xinjiang) | 82.17383 | 44.44353 | 640 |
| var. tomortensis |  |  |  |  |
| WENS | Wensu(Xinjiang) | 80.57537 | 41.76357 | 640 |
| ***P. pilosa*** |  |  |  |  |
| BEIT | Beitun(Xinjiang) | 87.87383 | 47.34839 | 497 |
| HAB | Habahe(Xinjiang) | 86.34775 | 48.07620 | 526 |
| BUE | Buerjin(Xinjiang) | 86.83521 | 47.71557 | 471 |
| var. *leiocarpa* |  |  |  |  |
| WENS | Wensu(Xinjiang) | 80.57537 | 41.76357 | 2355 |
| ***P. pamirica*** |  |  |  |  |
| AK | Aketao(Xinjiang) | 75.52342 | 38.98026 | 1773 |

**Table S2.** Summary of the statistics of Genome sequencing data for 87 samples analyzed in this study. The individuals for which genome sequences were downloaded from the Genome Sequence Archive (GSA) are marked by “*”, while those downloaded from the NCBI are marked by “#”.

| Species | Locality | **Source/Accession No.** | **data size（bp）** | **Collection No.** |
| --- | --- | --- | --- | --- |
| *P. laurifolia* JIM | Xinjiang, China | This study | 8,451,586,689 | WXY057 |
| *P. laurifolia* EM | Xinjiang, China | This study | 7,813,244,697 | WXY083 |
| *P. laurifolia* HEB | Xinjiang, China | This study | 8,892,204,121 | WXY066 |
| *P. laurifolia* HAB | Xinjiang, China | This study | 8,862,021,722 | WXY018 |
| *P. laurifolia* BUE | Xinjiang, China | This study | 8,168,254,841 | WXY029 |
| *P. laurifolia* FUY | Xinjiang, China | This study | 11,799,524,906 | WXY047 |
| *P. laurifolia* AL | Xinjiang, China | This study | 14,302,271,323 | ZCL8D |
| *P. laurifolia* BEIT | Xinjiang, China | This study | 13,086,201,189 | XS37 |
| *P. laurifolia* QINGH | Xinjiang, China | This study | 5,771,128,466 | I”-4169 |
| *P. laurifolia* FUH | Xinjiang, China | This study | 7,836,717,039 | I”-3925 |
| *P. talassica* KUC | Xinjiang, China | This study | 13,689,926,560 | WXY241 |
| *P. talassica* XINY | Xinjiang, China | This study | 10,340,768,007 | WXY206 |
| *P. talassica* WUS | Xinjiang, China | This study | 9,652,921,859 | WXY446 |
| *P. talassica* HUOC | Xinjiang, China | This study | 11,802,932,055 | WXY160 |
| *P. talassica* GONGL | Xinjiang, China | This study | 5,265,626,911 | WXY194 |
| *P. talassica* JINGH | Xinjiang, China | This study | 8,196,883,357 | WXY133 |
| *P. talassica* WENS | Xinjiang, China | This study | 8,555,612,105 | WXY291 |
| *P. talassica* WUL1 | Xinjiang, China | This study | 7,984,842,303 | WP0101 |
| *P. talassica* WUL2 | Xinjiang, China | This study | 7,268,680,690 | WP0102 |
| *P. talassica* WUL3 | Xinjiang, China | This study | 13,585,150,285 | WP0205 |
| *P. talassica* WUL4 | Xinjiang, China | This study | 11,450,419,180 | WP0210 |
| *P. talassica* FUK1 | Xinjiang, China | This study | 7,268,680,690 | WP0305 |
| *P. talassica* FUK2 | Xinjiang, China | This study | 9,359,493,883 | WP0308 |
| *P. talassica* FUK3 | Xinjiang, China | This study | 7,348,762,208 | WP0507 |
| *P. talassica* FUK4 | Xinjiang, China | This study | 12,253,141,592 | WP0501 |
| *P. talassica* FUK5 | Xinjiang, China | This study | 8,753,742,149 | WP0408 |
| *P. talassica* FUK6 | Xinjiang, China | This study | 9,624,802,057 | WP0406 |
| *P. talassica* QIT1 | Xinjiang, China | This study | 6,635,409,782 | WP0601 |
| *P. talassica* QIT2 | Xinjiang, China | This study | 8,054,037,606 | WP0609 |
| *P. talassica* QIT3 | Xinjiang, China | This study | 10,855,188,543 | WP0703 |
| *P. talassica* QIT4 | Xinjiang, China | This study | 10,347,968,327 | WP0706 |
| *P. talassica* CHANGJ | Xinjiang, China | This study | 7,293,949,115 | WP0809 |
| *P. talassica* MAN1 | Xinjiang, China | This study | 8,340,286,929 | WP0902 |
| *P. talassica* MAN2 | Xinjiang, China | This study | 10,088,088,866 | WP0903 |
| *P. talassica* var. *cordata*1 | Xinjiang, China | This study | 8,489,496,533 | WXY149 |
| *P. talassica* var*. cordata*2 | Xinjiang, China | This study | 9,019,697,636 | WXY144 |
| *P. pilosa* var. leiocarpa | Xinjiang, China | This study | 10,457,744,862 | WXY294 |
| *P. talassica* var. tomortensis | Xinjiang, China | This study | 7,084,777,126 | WXY304 |
| *P. pilosa* HAB | Xinjiang, China | This study | 9,074,230,371 | WXY008 |
| *P. pilosa* BUE | Xinjiang, China | This study | 7,150,751,752 | WXY024 |
| *P. pilosa* BEIT1 | Xinjiang, China | This study | 9,581,489,249 | WXY003 |
| *P. pilosa* BEIT2 | Xinjiang, China | This study | 9,454,975,911 | WXY001 |
| *P. pamirica* AK1 | Xinjiang, China | This study | 9,516,478,748 | WXY351 |
| *P. pamirica* KNP# | Khunjerab National Park(Pakistan) | SAMN17141139 | 6,167,730,886 | ppam1/FPH368 |
| *P. pamirica* AK2# | Xinjiang, China | SAMN17141159 | 4,554,166,224 | ppam3/Y177-1 |
| *P. talassica* WUL# | Xinjiang, China | SAMN17141145 | 3,673,586,359 | ptal1/I-899 |
| *Populus euphratica*# | Xinjiang, China | SAMN17141146 | 4,846,822,977 | peup1 |
| *Populus adenopoda** | Xinjiang, China | CRX045735 | 4,097,646,046 | pade1 |
| *Populus alba*# | Xinjiang, China | SAMN17141177 | 2,585,004,465 | palb1 |
| *Populus davidiana** | Henan, China | CRX045742 | 2,861,506,593 | pdav1 |
| *Populus tremula#* | Xinjiang, China | SAMN17141176 | 2,793,118,321 | ptre1 |
| *Populus heterophylla* 1# | Illinois, USA | SAMN17141151 | 5,247,299,737 | pwil1/Y009 |
| *Populus heterophylla* 2# | New York, USA | SAMN17141152 | 4,681,648,414 | pwil2/Y134-1 |
| *Populus lasiocarpa* 1# | Sichuan, China | SAMN17141192 | 2,419,874,169 | phet1/ LS20190721-03 |
| *Populus glauca* 1# | Xizang, China | SAMN17141164 | 3,425,515,682 | plas1/I-1137 |
| *Populus fremontii* 1* |  | CRX045770 | 13,764,395,817 |  |
| *Populus deltoides* 1# | New York, USA | SAMN17141105 | 2,281,914,724 | pdel3/6162 |
| *Populus deltoides* 2# | Montana, USA | SAMN17141106 | 1,961,336,737 | pdel4/11133 |
| *Populus iliensis#* | Xinjiang, China | SAMN17141158 | 4,125,370,726 | pilie1/Y163-1 |
| *Populus nigra* 3# | Xinjiang, China | SAMN17141143 | 26,422,898,944 | I-738 |
| *Populus* × *jrtyschensis* 2# | Xinjiang, China | SAMN17141113 | 14,375,479,964 | Y248 |
| *Populus afghanica* AK | Xinjiang, China | This study | 16,306,927,235 | WXY-325 |
| *Populus afghanica* TAS | Xinjiang, China | This study | 10,521,307,423 | WXY-370 |
| *Populus afghanica* 1# | Xinjiang, China | SAMN17141165 | 2,716,043,698 | pafg1/ Y182-24 |
| *Populus nigra* HAB | Xinjiang, China | This study | 8,834,959,847 | WXY-011 |
| *Populus nigra* FUH | Xinjiang, China | This study | 11,988,116,633 | WXY-049 |
| *Populus trinervis* 1# | Sichuan, China | SAMN17141125 | 3,772,139,555 | ptrin1/ PH20120611-04 |
| *Populus trinervis* 2# | Gansu, China | SAMN17141126 | 4,130,849,510 | ptrin2/ I-2501 |
| *Populus balsamifera* 2# | Maine, USA | SAMN17141190 | 3,149,812,011 | pbal5/ 6436 |
| *Populus trichocarpa* 1# |  | SAMN17141180 | 2,713,976,275 | ptric1/ 19698218C |
| *Populus rockii* 2# | Shannxi, China | SAMN17141174 | 4,370,172,213 | FP-8/ Y082 |
| *Populus xiangchengensis* 1# | Sichuan, China | SAMN17141128 | 3,818,734,474 | pxia2/ 11-0290 |
| *Populus szechuanica* 2# | Yunnan, China | SAMN17141140 | 3,829,540,249 | pyun1/ PH20120327-12 |
| *Populus ciliata#* | Xizang, China | SAMN17141134 | 2,875,412,202 | pcil1/ Y30-2 |
| *Populus haoana* 1# | Yunnan, China | SAMN17141167 | 2,793,633,260 | phao2/ I-66 |
| *Populus pseudoglauca* 1# | Xizang, China | SAMN17141168 | 1,947,559,390 | ppgla1/ PH20120905-02 |
| *Populus suaveolens* 1# | Heilongjiang, China | SAMN17141150 | 4,819,110,975 | psua1/ P1829/I-180 |
| *Populus cathayana* 1# | Shannxi, China | SAMN17141127 | 2,694,947,426 | pcat1/ Y121 |
| *Populus nigra* 2# | Xinjiang, China | SAMN17141114 | 5,809,387,693 | I-1231 |
| *Populus nigra* Neitherlands# | Neitherlands | SAMN04299914 |  | NL-2051 |
| *Populus nigra* Spain# | Spain | SAMN04299909 | 10,854,942 | C1 |
| *Populus nigra* Italy1# | Italy | SAMN04299908 |  | SN-26 |
| *Populus nigra* Hungary# | Hungary | SAMN04299905 |  | FTNY19 |
| *Populus nigra* Italy# | Italy | SAMN04299902 |  | SN-11 |
| *Populus nigra* France2# | France | SAMN04299901 |  | 98568-1 |
| *Populus nigra* France1# | France | SAMN04299894 |  | 6-A31 |
| *Populus nigra* Germany# | Germany | SAMN04299893 |  | NVHOF2/19 |

**Table S3.** Morphological comparison of short branch leaves among *P. laurifolia* complex species. Measurements include: leaf length (LL); leaf width (LW); leaf width-to-length ratio (LWR); leaf base angle (ALB); ratio of the distance from the widest point to the leaf base to the leaf length (WLR); position of the widest point of the leaf blade (WLP); petiole length (PL); petiole length-to-leaf length ratio (PLR); and density of petiole (DPP) pubescence among *P. laurifolia* complex species.

| **Index name** | | ***P. laurifolia*** | ***P. pilosa*** | ***P. talassica*** | ***P. talassica* var. cordata** | **P. talassica var.**  **tomortensis** | ***P. pamirica*** |
| --- | --- | --- | --- | --- | --- | --- | --- |
| LL(mm) | Mean±SE  Minimum-Maximum | 89.70±0.93  53.01-137.59 | 71.37±2.72  40.17-104.97 | 80.04±0.69  39.33-133.34 | 75.17±1.54  49.82-95.64 | 78.01±2.98  56.90-109.92 | 77.54±2.20  39.74-106.04 |
| LW(mm) | Mean±SE  Minimum-Maximum | 54.6225±0.61  34.31-94.82 | 50.73±2.42  25.90-83.55 | 49.90±0.50  23.44-94.34 | 52.13±1.11  34.09-65.12 | 53.38±1.64  30.09-66.46 | 68.93±2.46  41.13-105.49 |
| LWR(%) | Mean±SE  Minimum-Maximum | 61.47±5.23  41.00-87.00 | 70.54±1.56  50.00-92.00 | 62.43±0.35  36.00-88.00 | 69.54±0.92  56.00-84.00 | 69.43±1.93  37.00-83.00 | 89.39±2.10  57.00-119.00 |
| ALB(°) | Mean±SE  Minimum-Maximum | 106.43±0.96  63.31-139.81 | 79.10±2.24  49.34-110.41 | 103.95±0.72  52.51-161.74 | 80.70±1.53  62.14-103.74 | 94.89±3.56  56.98-126.66 | 93.58±3.30  54.63-138.81 |
| WLP(mm) | Mean±SE  Minimum-Maximum | 29.88±0.44  10.19-56.64 | 23.32±1.10  49.34-110.41 | 27.77±0.25  13.23-50.13 | 23.01±0.59  14.32-29.52 | 26.79±1.68  17.12-44.90 | 28.86±0.83  11.63-39.24 |
| WLR(%) | Mean±SE  Minimum-Maximum | 33.19±0.31  20.02-46.59 | 32.36±0.65  19.00-40.00 | 34.90±0.20  22.00-51.00 | 30.53±0.38  26.00-38.00 | 33.79±0.87  28.00-42.00 | 37.40±0.68  29.00-45.03 |
| PL(mm) | Mean±SE  Minimum-Maximum | 36.98±0.62  10.60-68.06 | 31.73±1.14  15.11-52.91 | 35.43±0.50  4.29-74.13 | 44.00±1.39  30.42-69.69 | 36.90±2.33  15.21-59.55 | 55.71±2.13  18.93-64.11 |
| PLR(%) | Mean±SE  Minimum-Maximum | 41.64±0.65  9.00-77.00 | 45.19±1.92  3.00-76.00 | 44.15±0.50  7.00-74.00 | 58.43±1.20  44.00-73.00 | 46.92±2.20  24.00-64.00 | 71.81±1.97  45.00-99.00 |
| DPP(hairs/mm²) | Mean±SE  Minimum-Maximum | 102.73±8.07  7.00-312.00 | 106.96±12.57  29.00-261.00 | 9.24±1.34  0.00-177.00 | 1.19±0.71  0.00-10.00 | 61.63±12.95  21.00-114.00 | 163.00±7.02  155.00-177.00 |

**Table S4.** Morphological comparison of sprout leaves among *P. laurifolia* complex species. Measurements include: leaf length (LL); leaf width (LW); leaf width-to-length ratio (LWR); leaf base angle (ALB); ratio of the distance from the widest point to the leaf base to the leaf length (WLR); position of the widest point of the leaf blade (WLP); petiole length (PL); and petiole length-to-leaf length ratio (PLR) among *P. laurifolia* complex species.

| **Index name** | | ***P. laurifolia*** | ***P. pilosa*** | ***P. talassica*** | ***P. pamirica*** |
| --- | --- | --- | --- | --- | --- |
| LL(mm) | Mean±SE  Minimum-Maximum | 86.0221±5.06  53.51-125.69 | 83..93±2.18  74.44-89.01 | 85.72±3.133  60.11-127.06 | 73.56±2.44  61.76-82.50 |
| LW(mm) | Mean±SE  Minimum-Maximum | 25.10±1.43  14.78-37.19 | 20.22±1.09  14.97-23.73 | 30.25±1.19  20.83-45.31 | 21.77±1.68  15.87-28.74 |
| LWR(%) | Mean±SE  Minimum-Maximum | 29.63±1.10  22.00-39.00 | 24.10±0.87  20.00-27.00 | 37.04±0.83  31.00-46.00 | 29.44±1.17  22.00-35.00 |
| ALB(°) | Mean±SE  Minimum-Maximum | 122.48±4.06  102.98-158.96 | 151.39±1.87  145.76-159.21 | 114.14±3.93  90.00-151.92 | 138.13±3.69  120.23-154.21 |
| WLP(mm) | Mean±SE  Minimum-Maximum | 39.96±2.80  25.84-71.41 | 41.69±2.23  34.00-52.61 | 26.64±1.94  11.22-50.42 | 33.95±1.47  28.86-43.82 |
| WLR(%) | Mean±SE  Minimum-Maximum | 47.11±2.18  27.00-66.00 | 49.74±1.81  45.00-59.00 | 31.62±2.24  14.00-61.00 | 46.62±2.62  37.00-58.00 |
| PL(mm) | Mean±SE  Minimum-Maximum | 5.32±0.78  1.49-12.74 | 2.41±0.28  1.48-3.39 | 9.10±1.05  1.75-24.18 | 5.28±1.28  1.66-12.56 |
| PLR(%) | Mean±SE  Minimum-Maximum | 5.80±0.63  2.00-11.00 | 2.88±3.32  2.00-4.00 | 10.44±0.93  2.00-22.00 | 6.94±1.53  2.00-15.00 |
