# Supplementary material for: Species delimitation in the Populus laurifolia complex (Salicaceae) based on phylogenetic and morphometric evidence
Source: Front Plant Sci. 2025 Feb 6;16:1518122. doi: 10.3389/fpls.2025.1518122 (PMC11839596; doi:10.3389/fpls.2025.1518122)
Supplement: Supplementary file 2 [file DataSheet1.docx]

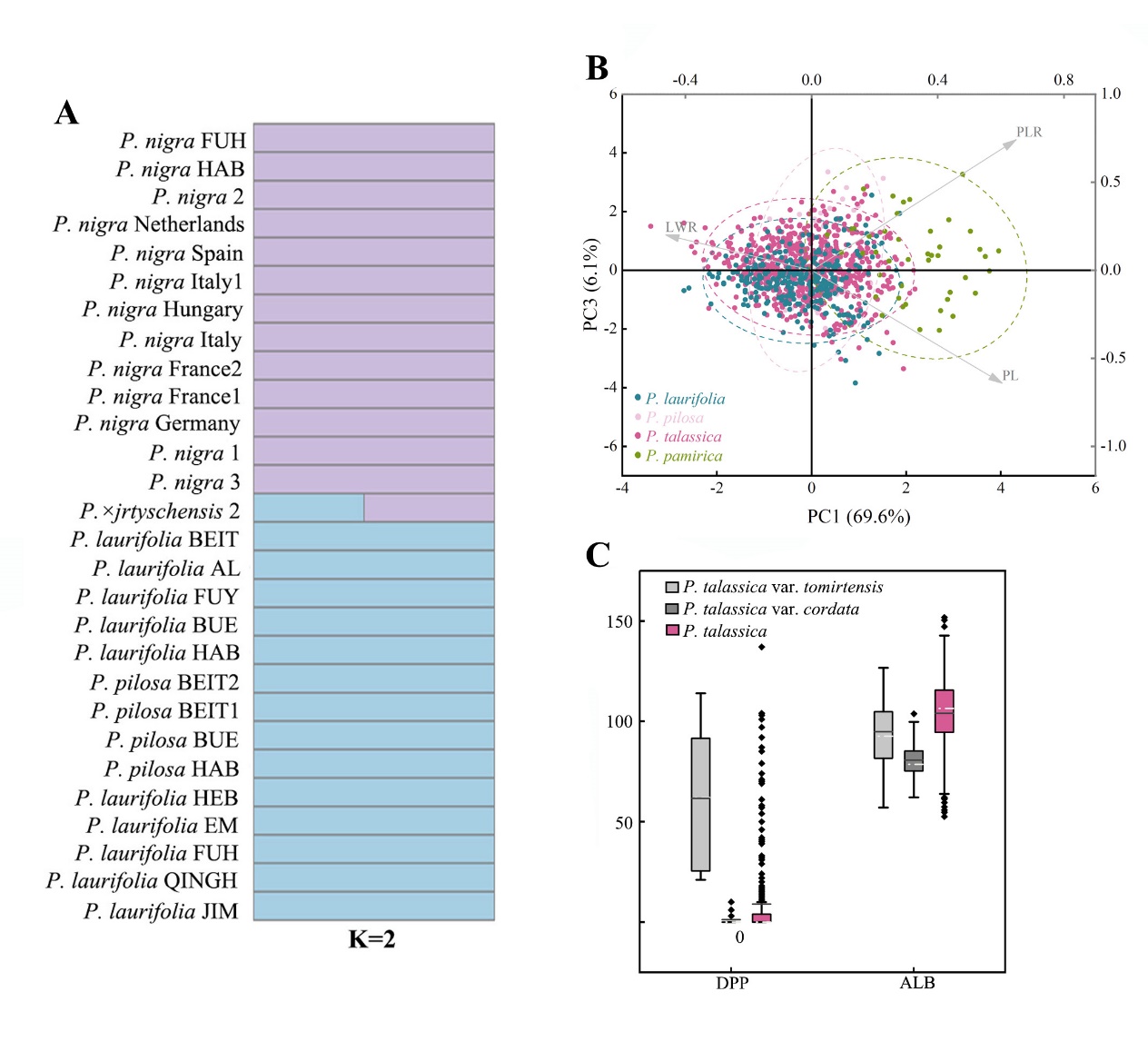


**Figure S1.** (A) Population structure analysis for *P. laurifolia*, *P. nigra* and *Populus × jrtyschensis*. (B) Principal Component Analysis (PCA) based on morphological data of short branch leaves. Species are represented by different colors: blue for *P. laurifolia*, light pink for *P. pilosa*, red for *P. talassica*, and green for *P. pamirica*. ALB: leaf base angle, DPP: density of petiole pubescence. (C) Morphological comparison of key distinguishing features among *P. talassica* and its two varieties.


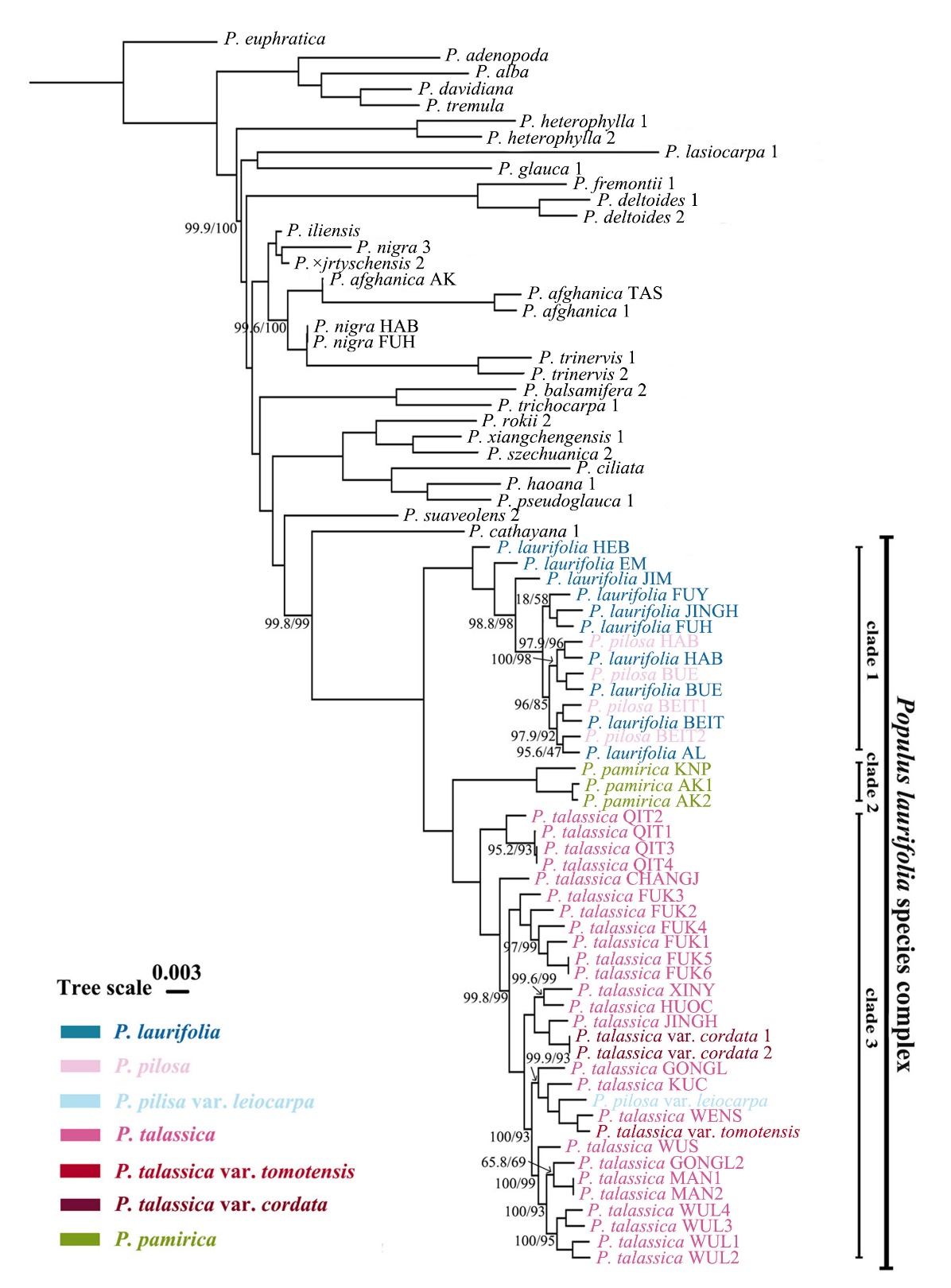


**Figure S2.** Phylogenetic relationship of the *Populus laurifolia* species complex reconstructed by IQ-TREE based on 566,375 nuclear SNPs. Unless otherwise indicated, all nodes had 100% supports of SH-aLRT bootstrap (Alrt) and Ultrafast bootstrap (UFBoot).


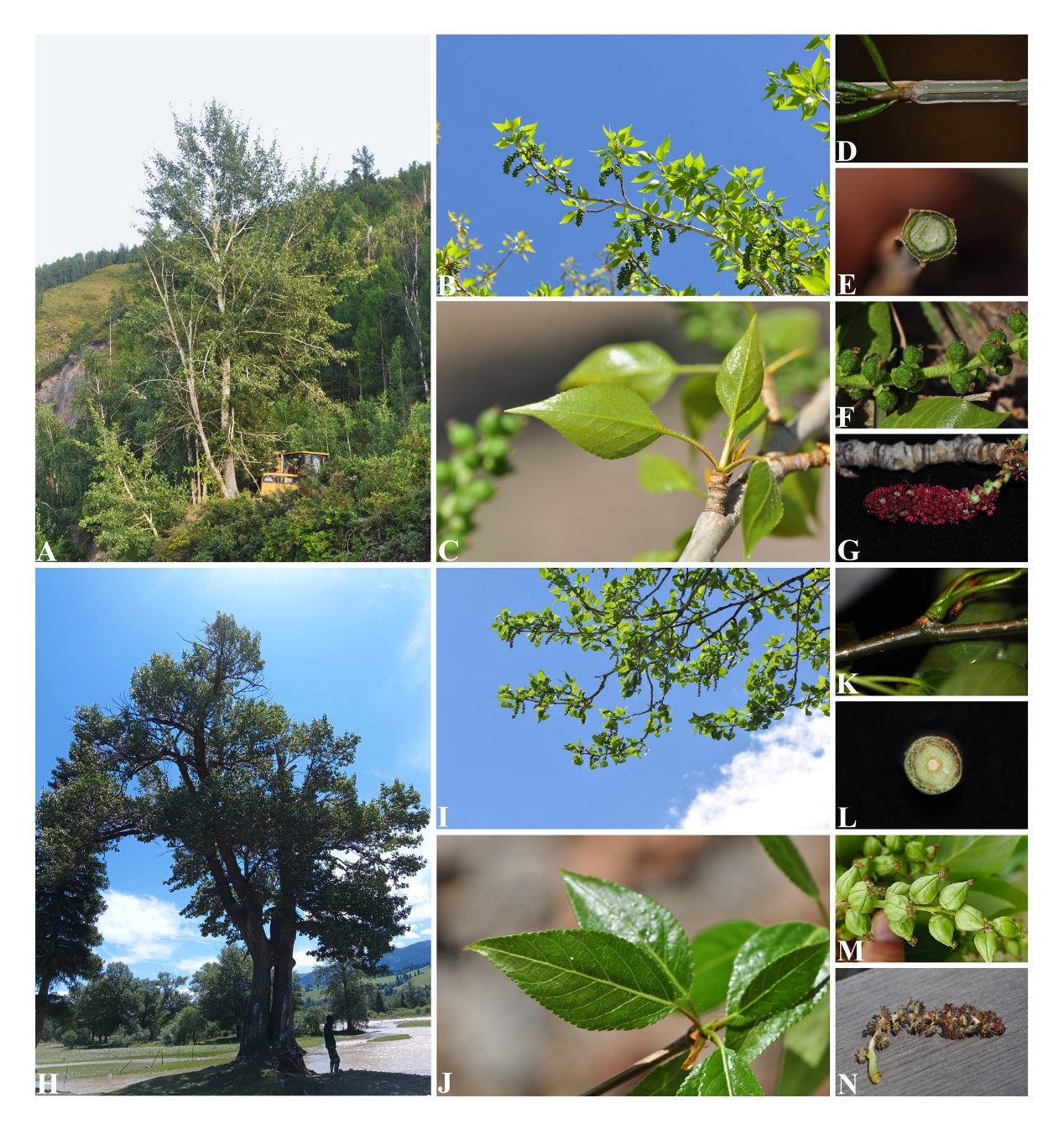


**Figure S3.** Photographic images of *P. laurifolia* (A, B, C, D, E, F, G) and *P. talassica* (H, I, J, K, L, M, N).


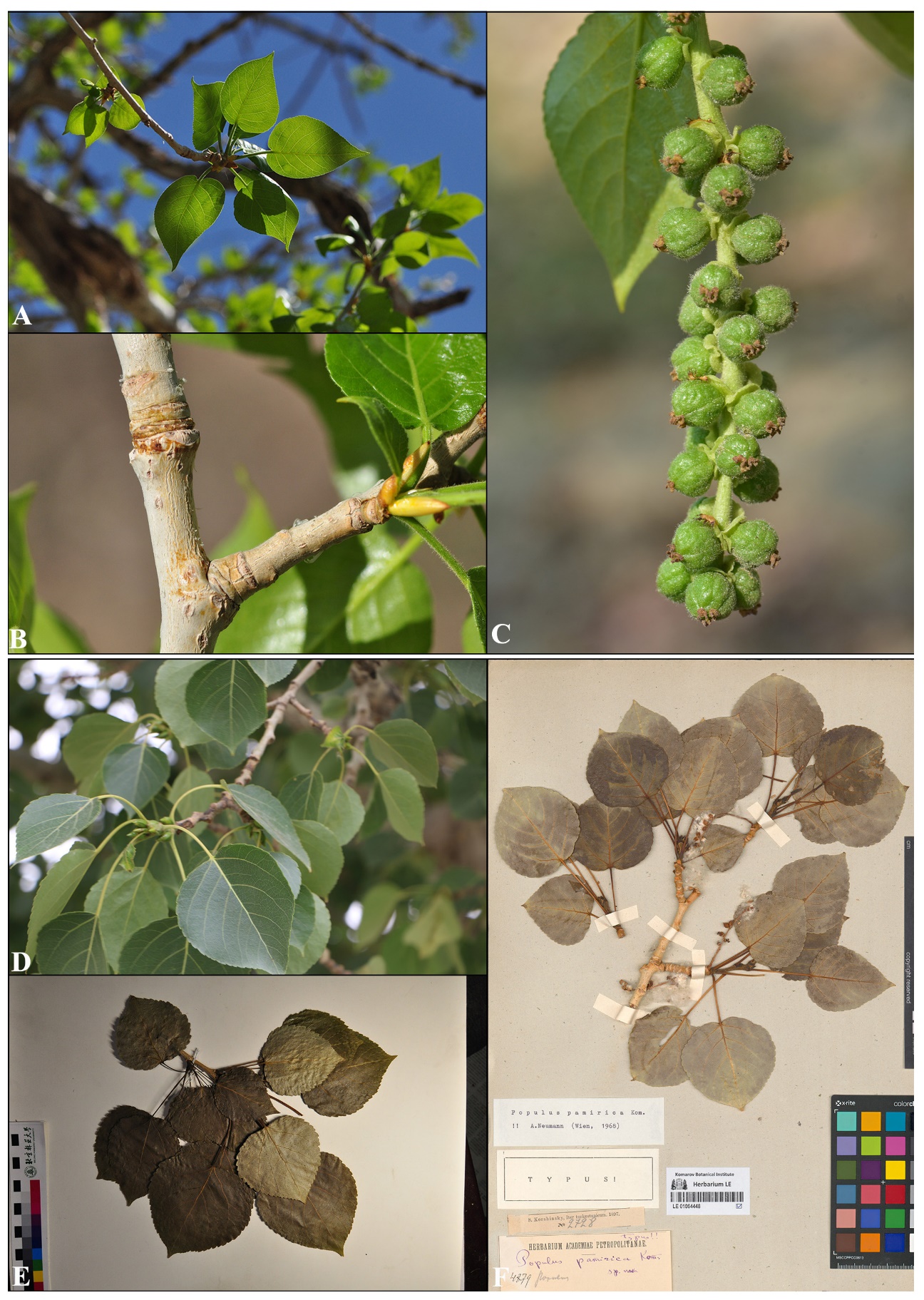


**Figure S4.** Morphological Characteristics of *P. pilosa* (A, B, C) and *P. pamirica* (D, E, F). (A) Leaves of *P. pilosa*. (B) Branches of *P. pilosa*. (C) Fruits of *P. pilosa*. (D) type specimen of *P. pamirica*. (E, F) leaves of *P. pamirica*.


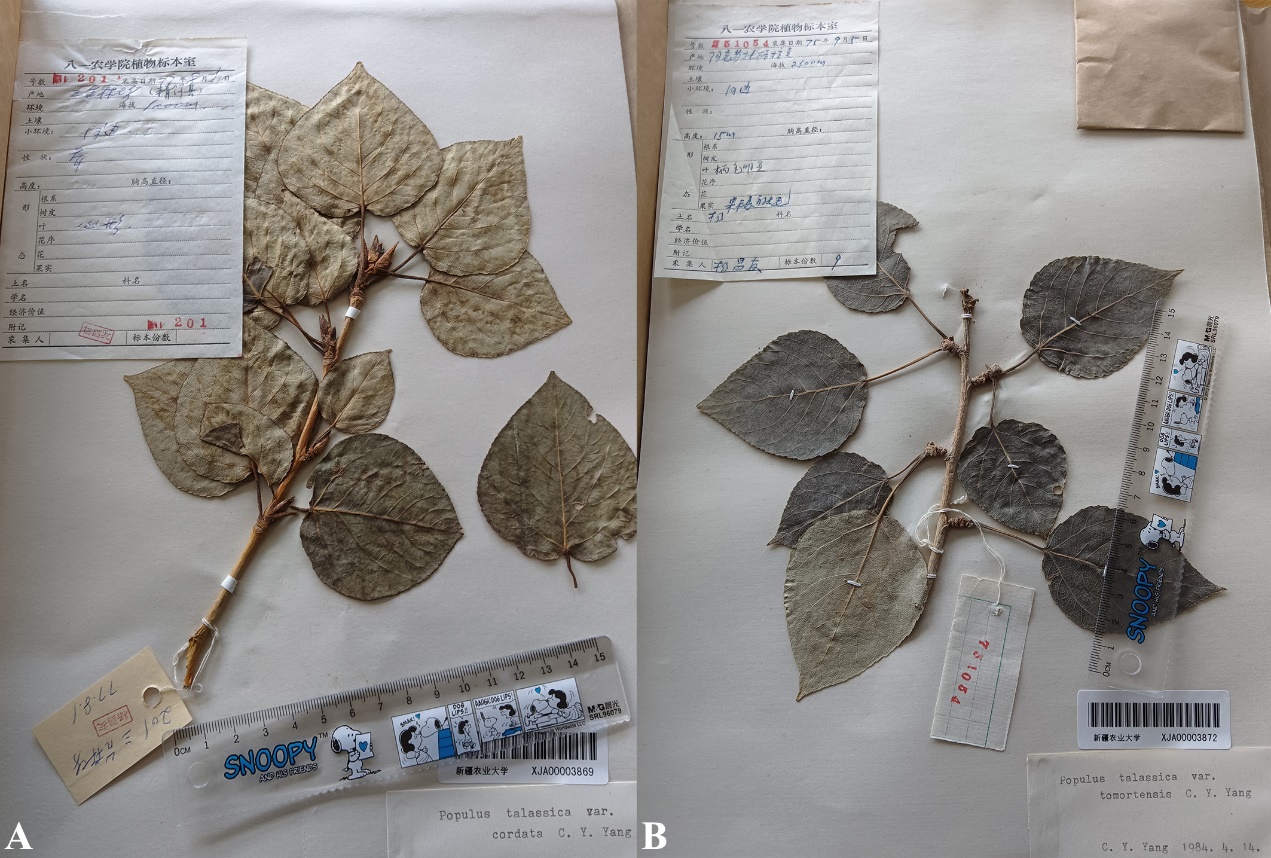


**Figure S5.** type specimens of (A) *P. talassica* var. *cordata* and (B) *P. talassica* var. *tomortensis.*


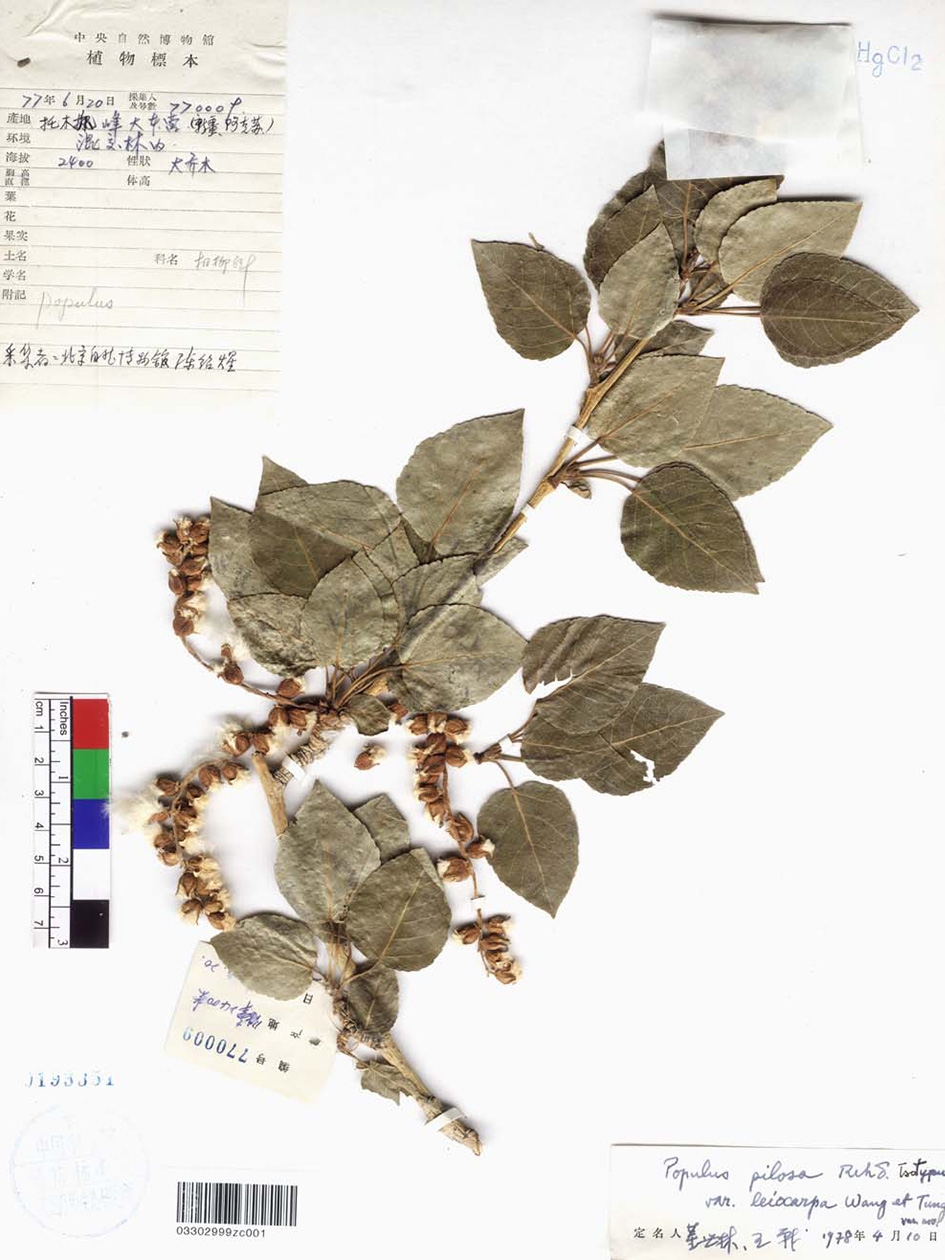


**Figure S6.** type specimen of *P. pilosa* var. *leiocarpa.*
